# Catalytic Enantioselective Rauhut–Currier Reaction Mediated by Lithium Selenolates

**DOI:** 10.1021/acsomega.4c08290

**Published:** 2025-03-18

**Authors:** Gabriela Całka-Kuc, Seweryn Żubrowski, Szymon Buda

**Affiliations:** † Faculty of Chemistry, Jagiellonian University, Gronostajowa 2, 30-387 Krakow, Poland; ‡ Doctoral School of Exact and Natural Sciences, Jagiellonian University, Łojasiewicza 11, 30-348 Krakow, Poland

## Abstract

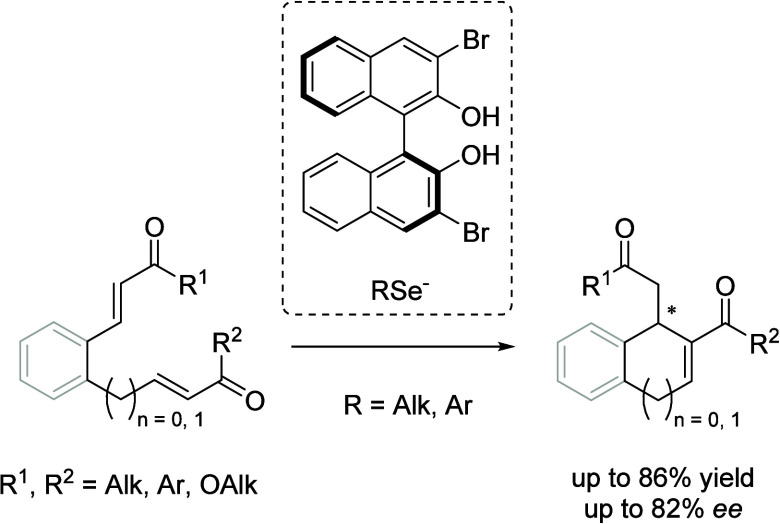

A catalytic enantioselective intramolecular Rauhut–Currier (IRC) reaction has been developed by using a combination of lithium selenolates and a catalytic amount (10 mol %) of an enantiopure BINOL ligand. The study focused particularly on evaluating the influence of BINOL ligands on the reaction’s outcome. The reaction conditions were optimized to achieve good yields and enantioselectivities. Screening of lithium organoselenolates identified phenyl lithium selenolate (PhSeLi) as the most efficient nucleophile. A variety of bis-α,β-unsaturated compounds were successfully cyclized under these conditions, demonstrating the broad applicability of the method. Detailed studies revealed the crucial role of water in the reaction and the importance of hydrogen bonding and Bro̷nsted acid catalysis in achieving asymmetric induction.

## Introduction

**Scheme 1:**
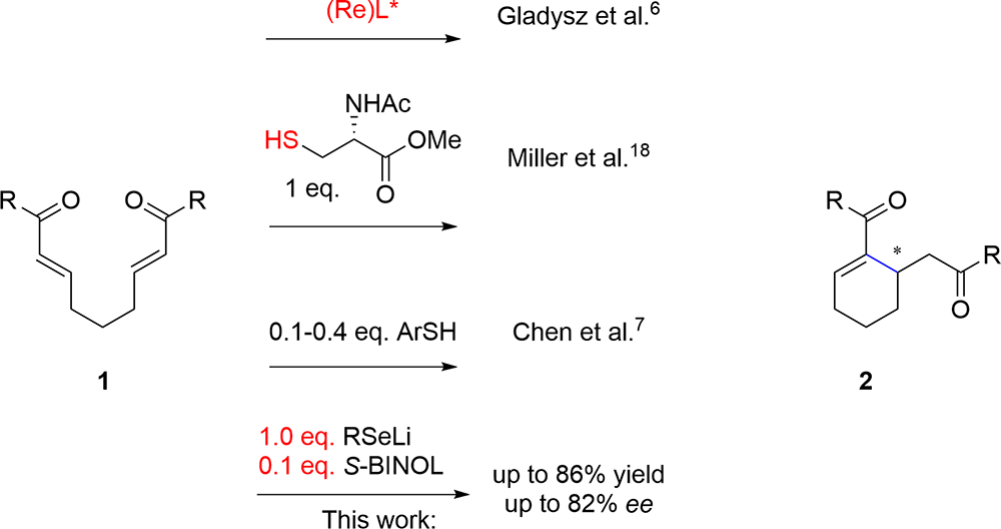
Examples of Asymmetric Intramolecular RC Reactions of Diverse Bisenone Substrates

The synthetic potential of organoselenium compounds has been established over the past three decades, and their broad utility is now recognized by organic chemists. Recently, a variety of chiral organoselenium catalysts have been developed, leading to extensive research in the asymmetric functionalization and intramolecular cyclization of alkenes.^1−7^ A simple combination of cyclohexaneselenol and a chiral NHC ligand can be an efficient tool for the enantioselective seleno-Michael reaction.^8^ Recently, the effective generation of magnesium-based selenium nucleophiles and seleno-Michael addition to α,β-unsaturated esters was presented by Wei and co-workers.^9^ The RC reaction has been recognized as a potentially useful organic reaction as it provides a one-step preparation of 2-methylene-1,5-dicarbonyl compounds from readily available α,β-unsaturated carbonyl compounds.^10,11^ The intramolecular version of the RC reaction offers a cyclic product under mild reaction conditions.^12,13^ The catalytic asymmetric modification of the intramolecular RC reaction has been of interest among organic chemists, and several reports have been published so far (Scheme 1).^14−18^

A preliminary report on the organoselenolate-mediated intramolecular Rauhut–Currier (IRC) reaction in the presence of 1 equiv of BINOL-Sc triflate complex is recently presented.^19^ This catalyst with lithium *n*-butylselenolate affects the enantioselectivity of the IRC reaction, resulting in moderate yields (up to 40%) and enantioselectivities (up to 80%) for the cyclic enones **2**. The development of a catalytic version of this selenium-mediated process would provide a valuable strategy for performing the enantioselective Rauhut–Currier reaction. In this paper, we present a realization of this idea using a chiral BINOL-lithium selenolate system. It is important to note that the catalytic system presented in this work does not contain a d-block metal cation. This paper reports a catalytic version of the asymmetric seleno-Michael/Michael domino reaction that offers a high yield and good *ee* values for the products.

## Results and Discussion

The investigation commenced with a re-examination of the stoichiometric variant of the IRC reaction (Table 1). The reaction of **1** with lithium *n*-butylselenolate in the presence of the (*S*)-BINOL-Sc(OTf)_3_ complex at −30 °C resulted in the formation of the cyclic intermediate with the *n*-BuSe fragment. Subsequent oxidation and elimination in the presence of 30% hydrogen peroxide yielded the expected product in 42% yield and 82% *ee*. When the reaction was performed in the presence of PTSA instead of water, the product was obtained in 56% yield with 92% *ee* (Table 1, entry 2) after an extended period of time (24 h). Subsequently, the reaction was repeated with a catalytic amount of various (*S*)-BINOL-metal salt complexes, including scandium(III), ytterbium(III), and europium(III) triflates. Finally, the reaction of **1** with *n*-BuSeLi in the presence of (*S*)-3,3′-dibromo-BINOL-Zr(O*t*Bu)_4_-NMI (20 mol %) formed the expected product **2** in a very low yield (less than 10%) with 26% *ee*.^20^ Similarly, both scandium(III) triflate (Table 1, entry 6) and zirconium(IV) *tert*-butoxide (Table 1, entry 5) under standard conditions presented low yields (<20%) with up to 40% ee. Considering the structure of the active chiral ligand, we selected (*S*)-3,3′-dibromo-BINOL for the further investigation to study the impact of metal salt, water, and base on the reaction efficiency and asymmetric induction (Table 1, entries 7–10). It was somewhat unexpected that a simple (*S*)-3,3′-dibromo-BINOL with 2 equiv of water gave the product in 49% yield with 42% *ee*. To the best of our knowledge, this represents the first example of asymmetric addition of lithium selenolate to a bis-α,β-unsaturated carbonyl compound, catalyzed by 20 mol % optically pure BINOL derivatives. This promising result was the starting point for the development of asymmetric catalytic intramolecular RC research.

**Table 1:**
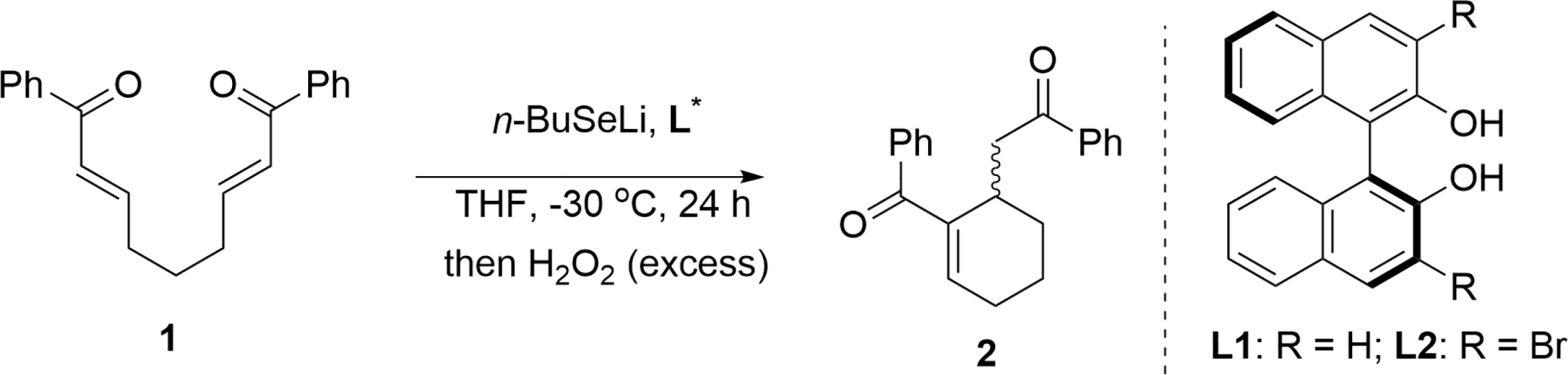
Preliminary Investigation of the Asymmetric IRC Reaction^a^

entry	conditions	yield [%]^b^	*ee* [%]^c^
1	1 equiv of **L1**, 1 eq Sc(OTf)_3_, 1.2 equiv of NMM, 10 equiv of water, 6 h	42	82
2	1 equiv of **L1**, 1 eq Sc(OTf)_3_, 1.2 equiv of NMM, 1 equiv of *p-*TsOH,	56	92
3	0.2 equiv of **L1**, 0.2 eq Sc(OTf)_3_, 0.24 equiv of NMM, 0.2 equiv of *p-*TsOH,	10	rac
4	0.2 equiv of **L2**, 0.2 eq Zr(O*t*Bu)_4_, 0.24 equiv of NMI, 0.2 equiv of *p-*TsOH,	<10	26
5	0.2 equiv of **L2**, 0.2 eq Zr(O*t*Bu)4, 0.24 equiv of NMI, 0.2 equiv of water	<10	20
6	0.2 equiv of **L2**, 0.2 eq Sc(OTf)_3_, 0.24 equiv of NMI, 0.2 equiv of water	<10	40
7	0.2 equiv of **L2**, 0.2 equiv of Sc(OTf)_3_, 0.2 equiv of water	nd	rac
8	0.2 equiv of **L2**, 0.24 equiv of NMI, 0.2 equiv of water	nd	rac
9	0.2 equiv of **L2**	35	rac
10	0.2 equiv of **L2**, 2 equiv of water	49	42

a Unless noted otherwise: Reactions were performed with 1 equiv of *n*-BuSeLi, THF, −30 °C, 24 h, then 1 mL of H_2_O_2_, −30 °C to RT. nd, not determined; rac, racemic mixture.

b Yield of the isolated product.

c Determined by HPLC analysis on a chiral stationary phase.

**Figure 1:**
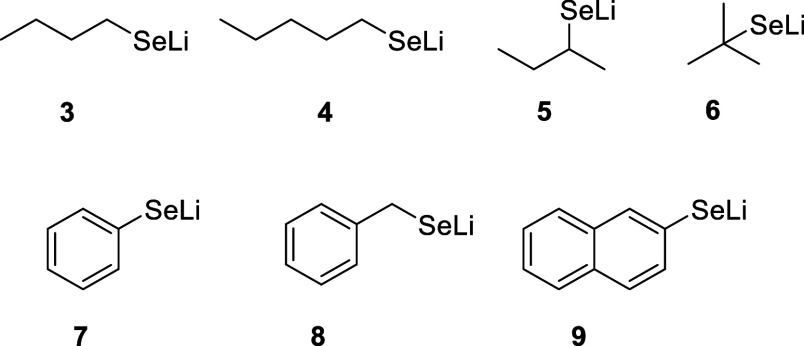
Structure of organoselenium nucleophiles **3**–**9**.

Based on the preliminary results, we investigated a series of lithium organoselenolates **3–9** (Figure 1). Lithium *n*-butyl (**3**) and *tert*-butyl-selenolate (**6**) were generated from elemental selenium and the corresponding organolithium reagent. The other lithium selenolates (**4**, **5**, and **7–9**) were prepared by treating the corresponding diselenide with *n*-BuLi.

Initial investigations revealed that the combination of *n*-BuSeLi with (*S*)-3,3′-dibromo-BINOL (**L2**) efficiently catalyzed the intramolecular RC reaction, resulting in the cyclic product in 49% yield (Table 2). In the reaction of 1 with linear selenolates (**3**–**4**), a moderate asymmetric induction (38–42% *ee*) was observed. Lithium *sec*-butylselenolate (**5**) produced the desired product with moderate *ee* and poor yield. When the reaction was performed in the presence of bulky selenolate **6**, the product was isolated in 64–65% yield and 60–62% *ee*. Lithium *tert*-butylselenolate was generated in two ways: from *tert*-BuLi and selenium (Table 2, entry 4) or from di-*tert*-butyl diselenide and *n*-BuLi (Table 2, entry 5). Phenyl lithium selenolate (**7**) was identified as the most efficient nucleophile in the formation of cyclic products. These results led to an improvement in the catalytic system, and thus, *tert*-BuSeLi and PhSeLi were advanced as nucleophilic sources of selenium for further studies.

**Table 2:**
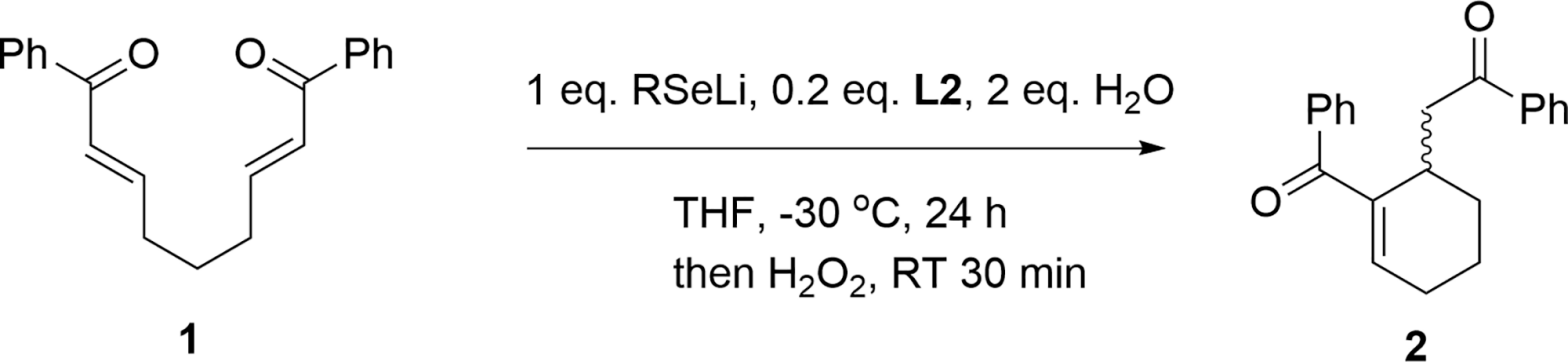
Screening of Organoselenium Nucleophiles

entry	selenolate	yield [%]^a^	*ee* [%]^b^
1	**3**	49	42
2	**4**	<10%	38
3	**5**	<10%	40
4	**6**	**64**	**60**
5	**6**	**65**	**62**
6	**7**	**85**	**80**
7	**8**	41	40
8	**9**	28	20

a Yield of the isolated product.

b Determined by HPLC analysis on a chiral stationary phase.

Having identified (*S*)-3,3′-dibromo-BINOL as a potential chiral additive, we conducted further optimizations, focusing on temperature, solvents, and equivalents of water (for a detailed description, please see the SI). After developing an efficient procedure for synthesizing the chiral cyclic product **2**, we examined the loading of chiral ligand **L2** (Table 3). The reaction of **1** with lithium *tert*-butylselenolate (**6**) in the presence of optically pure ligand **L2** at −30 °C for 24 h yielded the product in 68% yield and 60% *ee*. Switching the nucleophile from *tert*-butylselenolate to phenylselenolate (**7**) resulted in the desired product with 86% yield and 82% *ee*, even in a shorter reaction time (6 h). It is noteworthy that the combination of selenolate **7** and (*S*)-3,3′-dibromo-BINOL (**L2**) is highly enantioselective, even with a 1 mol % loading of the chiral ligand, providing the expected product in 77% yield and 56% *ee* (Table 3, entry 6).

**Table 3:**
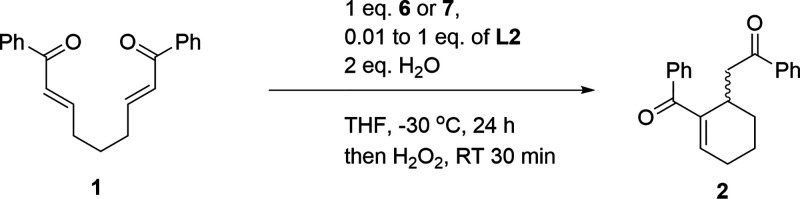
Influence of the Catalyst Loading

entry	nucleophile	time [h]	% of L2	yield [%]	*ee* [%]
1	**6**	24	5	62	40
2			10	**68**	**60**
3			20	65	62
4			50	50	40
5			100	57	70
6	**7**	6	1	77	56
7			5	84	72
8			10	**86**	**82**
9			20	82	80

**Scheme 2:**
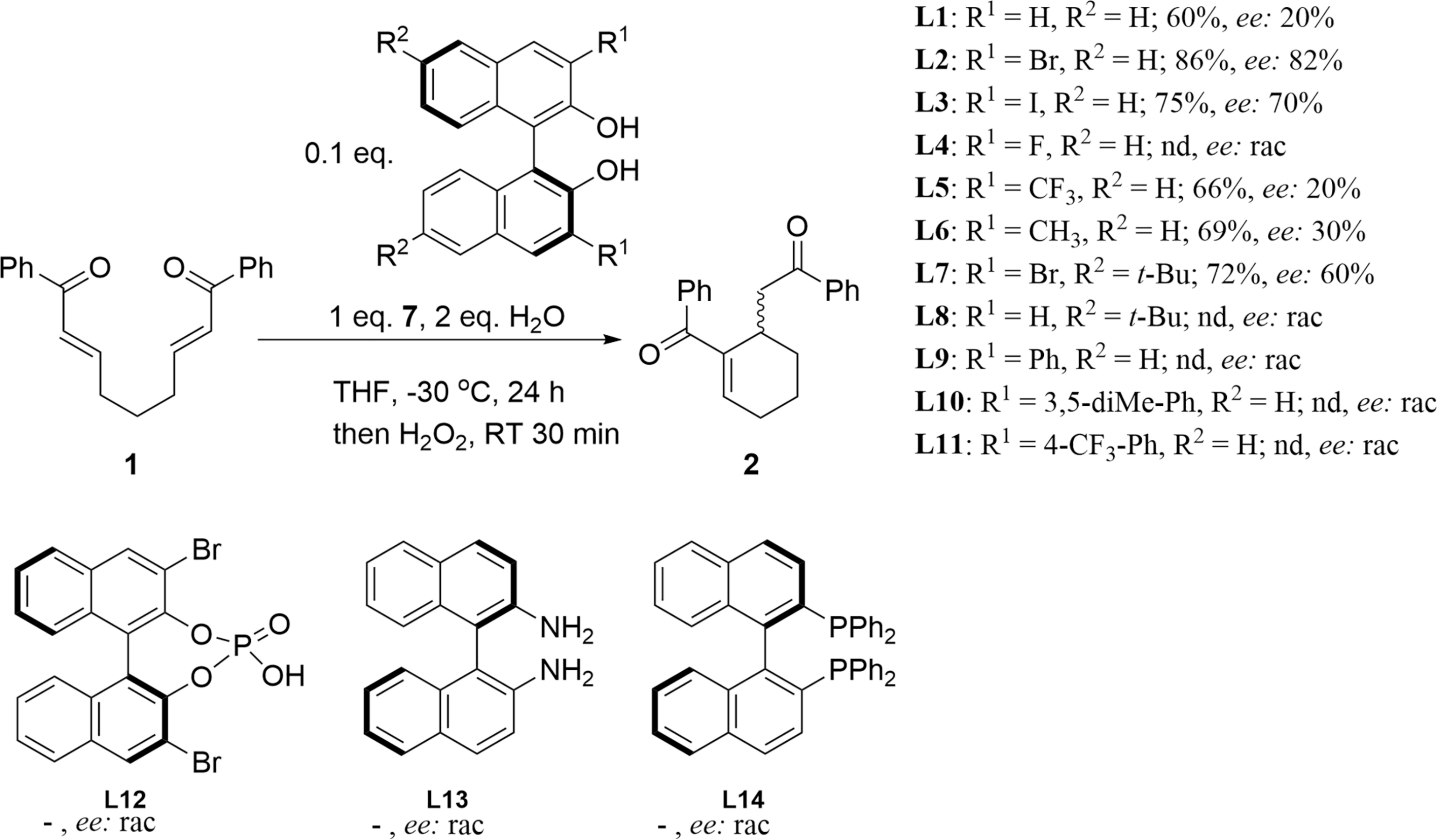
Investigation of Chiral BINOL Derivatives

Subsequently, we conducted a series of experiments involving the screening of different substituted (*S*)-BINOL and their derivatives with the phenyl lithium selenolate in an asymmetric intramolecular RC reaction of α,β-unsaturated ketones, induced with a nucleophilic selenium (Scheme 2). Upon testing various (*S*)-BINOL derivatives, it was observed that ligands with EWG groups, such as −Br (**L2**), −I (**L3**), and −CF_3_ (**L5**), exhibited greater activity in the reaction than did other ligands. Interestingly, ligand **L6**, which contains methyl groups, also demonstrated activity, resulting in the expected product with 69% yield and 30% *ee*. The simple (*S*)-BINOL (**L1**) resulted in 60% yield with 20% *ee*. The tetrasubstituted (*S*)-3,3′-dibromo-6,6’-di-*tert*-butyl-BINOL (**L7**) was active in the RC reaction, providing a high yield and good asymmetric induction of the desired product. In contrast, optically pure BINOL-phosphonate (**L12**) was not active. Binaphthyl amine (**L13**) and phosphine (**L14**) derivatives were found to be inactive under tested conditions. In the case of the remaining ligands (**L8**-**L11**), no asymmetric induction was observed. The aforementioned results indicated a correlation between the size of the 3,3′-position group and its electronic structure. Moreover, the substituent that enhanced the acidity of hydroxyl groups in the BINOL molecule led to a higher yield and superior enantiomeric excess of reaction products.^21^

**Scheme 3:**
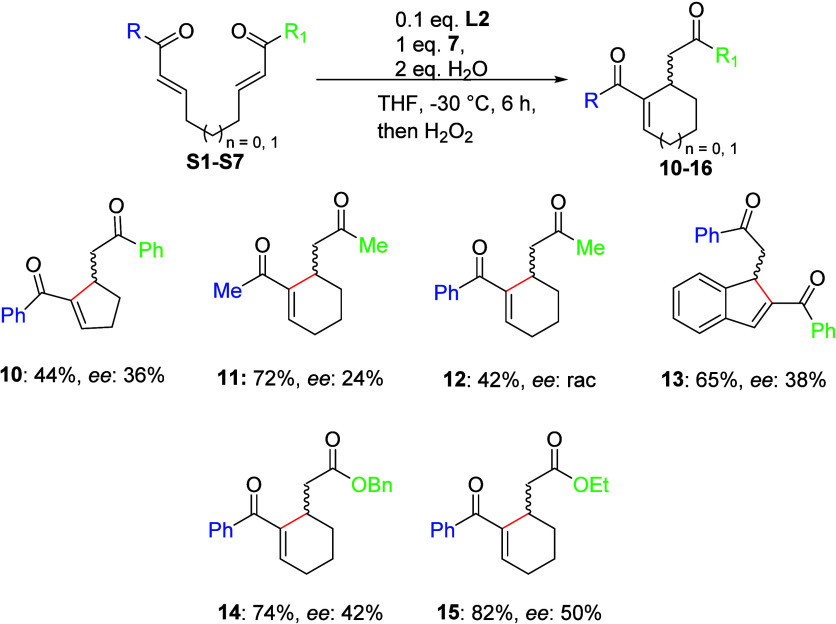
Selenium-Catalyzed Cyclization of Various Bis-α,β-Unsaturated Compounds

Finally, we evaluated the substrate scope of the selenolate-mediated enantioselective RC reaction (Scheme 3). For the synthesis of the substrate, please refer to the Supporting Information. The five-membered ring product (**10**) resulted in a lower yield and ee in comparison to the six-membered ring. Both aliphatic **S2** and mixed bisenone **S3** afforded their respective products (**11** and **12**) in good yields. Unexpectedly, the unsymmetrical ketobisenone **S3** yielded the anticipated product (**12**) in racemic form. The formation of the 1H-inden system (**13**) proceeded in satisfactory yield with moderate enantioselectivity. This fact can be explained by the effect of aromatic rings.

Finally, we turned our attention to mixed bisenone-enoate molecules (**S5***-***S7**) that are more challenging than bis-enones. The cyclization reaction proceeded smoothly with the ethyl ester (**14**, 74% yield, 42% *ee*) and the benzyl ester (**15**, 82% yield, 50% *ee*). No desired product was obtained in the reaction using the *tert*-butyl derivative (**S7**) due to steric hindrance. After the oxidation–elimination step, a loss of the high *E*/*Z* ratio in the α,β-unsaturated ketone fragment of **S7** was observed, as confirmed by LC–MS.

To gain more insight into the reaction mechanism, we performed several control experiments. Due to the rapid oxidation of organoselenium compounds, we were not able to isolate and analyze intermediates. When the reaction was conducted in the absence of selenolate in the presence of BINOL **L2**, the desired product was not formed, and the starting material was recovered (Table 4, entry 2). The p*K*_a_ for phenylselenol is reported to be 4.6^22^ or 5.9,^23^ depending on the source, while that for *tert*-butylselenol is 8.22,^22^ indicating that selenols are predominantly deprotonated to selenolates in the presence of phenolic hydroxyl groups. Li et al. determined the p*K*_a_ of (*S*)-3,3′-dibromo-BINOL (**L2**) to be 9.44 against 4-NO_2_-3-CF_3_-phenol as a pH indicator. According to our experimental data (Table 1, entry 9), water is crucial in the first stage of this reaction. Santi et al.^24^ observed that the addition of nucleophilic selenium (PhSeZnCl) to a Michael acceptor performed in the presence of water progresses 10–12× faster than in anhydrous THF. Moreover, selenols have improved stability in water than in THF.^25^ We assume that the asymmetric induction resulted from the Bro̷nsted acid catalysis and hydrogen bonds formed between BINOL and the substrate dictate the selectivity of this reaction.^26^ When the reaction proceeded in the presence of the lithium BINOL-ate (Table 4, entries 3 and 4) or methylated 3,3′-dibromo-BINOL **16** (Table 4, entry 5), the desired product formed as a racemic mixture. We carried out the reaction in the presence of 0.1 equiv of **L2** and 0.1 equiv of lithium phenylselenolate at standard temperature, which gave a product in poor yield (14%) but with excellent enantioselectivity up to 80% (Table 4, entry 6). Following that, we decided to run the control reaction with the same amount of chiral ligand and nucleophilic selenium at 80 °C. Those conditions yielded product **2** in 70% and 20% *ee* (Table 4, entry 7). We assume that the high temperature and the basic conditions cause a retro-seleno-Michael reaction and eliminate a phenylselenolate anion. This process regenerates a selenium nucleophile that can react with the next molecule of **1**.

**Table 4:**
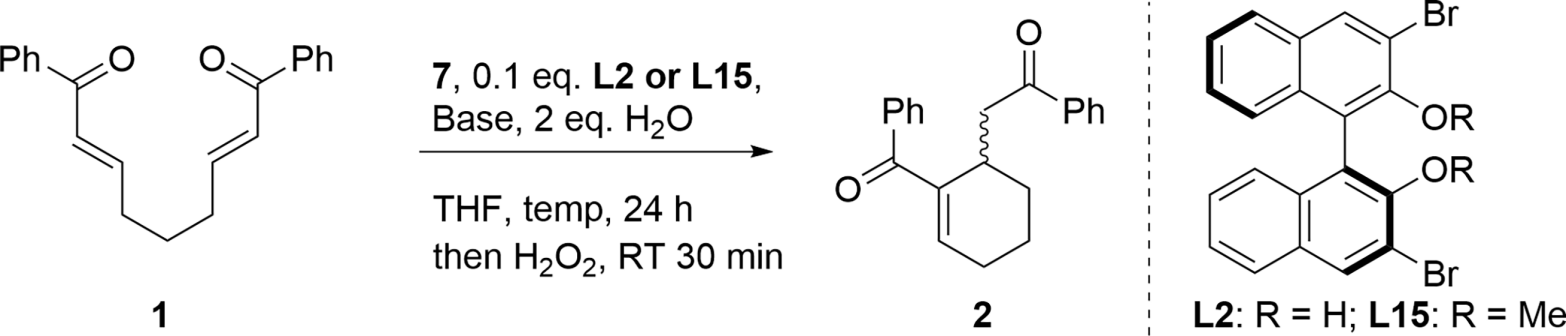
Control Experiments of the Intramolecular RC Reaction

entry	PhSeLi [eq]	ligand [eq]	base [eq]	temp [°C]	yield [%]	*ee* [%]
1	1.0 eq	**L2** (0.1eq)		–30	86	82
2	**--**	**L2** (0.1eq)		–30		a
3	1.0 eq	**L2** (1.0 equiv)	LiOH (0.2 equiv)	–30	nd	rac
4	1.0 eq	**L2** (1.0 equiv)	*n*-BuLi (0.2 equiv)	–30	nd	rac
5	1.0 eq	**L15** (0.1 equiv)		–30	nd	rac
6	0.1 eq	**L2** (0.1 equiv)		–30	14	80
7	0.1 eq	**L2** (0.1 equiv)		80	70	20^b^^,^^c^

a Starting material was recovered, no conversion.

b Reaction was performed in 1,4-dioxane without H_2_O_2_.

c Reaction time: 3 h.

**Scheme 4:**
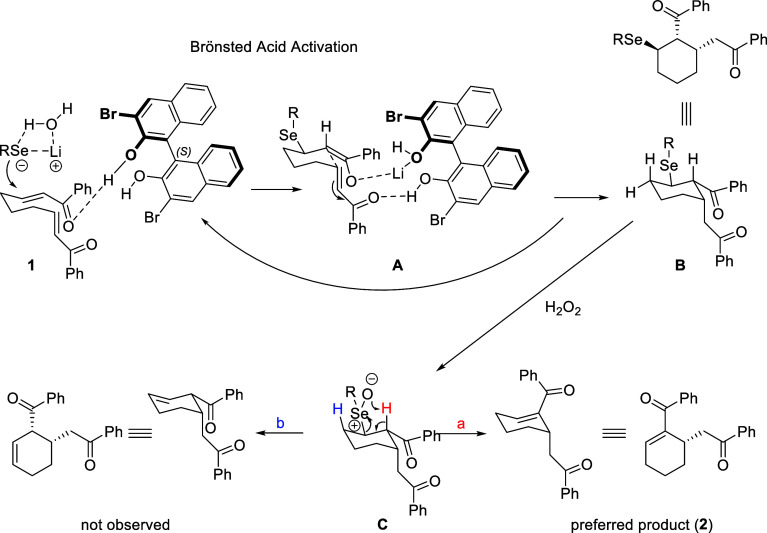
Plausible Reaction Mechanism Leading to α,β-Uunsaturated Cyclic Products

The reaction mechanism (Scheme 4) illustrates a Bro̷nsted acid activation pathway, leading to preferred product formation. The process begins with the activation of a substrate (**1**) by Bro̷nsted acid, which forms a stabilized intermediate complex (**A**) by coordination with lithium. Subsequent oxidation of intermediate **B** with hydrogen peroxide (H_2_O_2_) removes the selenium group via syn-selenoxide elimination. Intermediate **C** then undergoes regioselective elimination of PhSeOH, where pathway “a” leads to the formation of a more stable α,β-unsaturated product (**2**), while pathway “b”, which would form an alternative product, is not observed.

## Conclusions

In conclusion, our studies have successfully developed a catalytic enantioselective IRC reaction using a chiral BINOL-lithium selenolate system. Notably, this is the first reaction using a catalytic amount of BINOL in an asymmetric seleno-Michael addition. The optimized conditions provided high yields and good enantiomeric excesses for the cyclic products, particularly when PhSeLi was used as the nucleophile. Detailed investigation of the reaction pathway revealed the pivotal role of water and the nature of the asymmetric induction, highlighting the importance of hydrogen bonding and Bro̷nsted acid catalysis. This research represents a significant contribution to the field of organoselenium chemistry and asymmetric synthesis, providing a valuable strategy for the enantioselective Rauhut–Currier reaction.

## Experimental Section

### General Information

All of the starting materials and reagents were purchased from commercial sources and used without purification. Reactions were controlled using TLC on silica [aluminum plates (0.2 mm)]. The plates were visualized by UV light (254 nm). All reactions were performed under an argon atmosphere. Reaction products were purified by column chromatography using silica gel 60 (240–400 mesh). NMR spectra were recorded with a Bruker Advance 600 instrument. CDCl_3_ was used as a NMR solvent. ^1^H NMR spectra were recorded with 300 MHz and referenced relative to CDCl_3_–solvent residual peak (δ 7.26 ppm). Data are reported as follows: chemical shift in parts per million (ppm), multiplicity (s = singlet, d = doublet, t = triplet, dd = doublet of doublets, ddd = doublet of doublet of doublets, dddd = doublet of doublet of doublet of doublets, m = multiplet), coupling constants (in hertz), and integration. ^13^C NMR spectra were measured at 150 MHz with complete proton decoupling. HPLC analysis was performed on Knauer systems using a chiral column CHIRALPACK AD-H with UV detection.

#### General Procedure for the Rauhut–Currier Reaction

Procedure A: 13 mg of selenium (0.165 mmol) was placed into a vial, and 1 mL of dry and degassed THF was added. The mixture was cooled at 0 °C for 5 min, and then, 0.1 mL of *n*-BuLi (0.165 mmol, 1.6 M solution in hexane) was slowly added dropwise until the solution was discolored. Stirring was continued for another 5 min, and then 2 equiv of water and 10% of BINOL complex in 0.5 mL of THF were added dropwise. The reaction was then carried out under specified temperature conditions. After about 5 min, the appropriate starting material was added dropwise in 0.5 mL of THF by a syringe pump at a flow rate of 0.5 mL/min. After the specified time, 1 mL of H_2_O_2_ was added, and then, the mixture was allowed to warm to room temperature. After about 15 min, extraction with ethyl acetate was performed. The organic layers were collected and dried in anhydrous MgSO_4_. The solvent was evaporated, and the resulting oil was purified by column chromatography (hexane/ethyl acetate 6:1).

#### (*S*)-3,3′-dibromo-BINOL-Sc(OTf)_3_-NMI

To a suspension of Sc(OTf)_3_ (8 mg, 0.0165 mmol) in 0.3 mL of an anhydrous DCM, a solution of (*S*)-3.3′-dibromo-BINOL (7 mg, 0.0165 mmol) eq in 0.2 mL DCM) was added dropwise followed by NMI (36 μL, 0.033 mmol) in room temperature. After 30 min, the obtained complex was added dropwise to the previously generated selenolate with an appropriate proton source (water/PTSA). The reaction was then carried out under the specified temperature conditions. Further steps were performed according to the main procedure presented above.

#### (*S*)-3,3′- dibromo-BINOL-Zr(O*t*Bu)_4_-NMI

To a solution of Zr(O*t*Bu)_4_ (16 mg, 0.0165 mmol) and anhydrous DCM, (*S*)-3.3′-dibromo-BINOL (7 mg, 0.0165 mmol) in 0.2 mL of DCM was added dropwise, followed by NMI (36 μL, 0.033 mmol) at room temperature. After 30 min, the obtained complex was added dropwise to the previously generated selenolate with an appropriate proton source (water/PTSA). The reaction was then carried out under the specified temperature conditions. Further steps were performed according to the main procedure presented above.

Procedure B: 51 mg of Ph_2_Se_2_ (0.165 mmol) was placed into a vial, and 1 mL of dry and degassed THF was added. The mixture was cooled at 0 °C for 5 min, and then, 0.1 mL of *n*-BuLi (0.165 mmol, 1.6 M solution in hexane) was slowly added dropwise until the solution was discolored. Stirring was continued for another 5 min, and then, 2 equiv of water and 10% of (*S*)-3,3′-dibromo-BINOL (7 mg, 0.165 mmol) in 0.5 mL of THF were added dropwise. The reaction was then carried out under the specified temperature conditions. After about 5 min, the appropriate starting material was added dropwise in 0.5 mL of THF by a syringe pump at a flow rate of 0.5 mL/min. After the specified time, 1 mL of H_2_O_2_ was added, and then, the mixture was allowed to warm to room temperature. After about 15 min, extraction with ethyl acetate was performed. The organic layers were collected and dried over anhydrous MgSO_4_. The solvent was evaporated, and the resulting oil was purified by column chromatography (hexane/ethyl acetate 6:1).

##### 2-(2-Benzoylcyclohex-2-en-1-yl)-1-phenylethan-1-one (**2**)

A 50 mg portion of **1** (0.165 mmol) gave the product as a yellow oil (35 mg, 70%). The NMR shift values are consistent with previously reported data.^18^ Enantiomeric excess was determined by HPLC on a Chiralpak AD-H column, hexane/2-propanol = 96:4, flow rate = 0.5 mL/min; 21 °C; *t*_major_ = 15.14 min, *t*_minor_ = 20.69 min.

##### 2-(2-Benzoylcyclopent-2-en-1-yl)-1-phenylethan-1-one (**10**)

A 48 mg portion of **S1** (0.165 mmol) gave the product as a yellow oil (21 mg, 44%). The NMR shift values are consistent with previously reported data.^18^ Enantiomeric excess was determined by HPLC on a Chiralpak AD-H column, hexane: 2-propanol = 96:4, flow rate = 0.5 mL/min; 21 °C; *t*_major_ = 12.99 min, *t*_minor_ = 18.26 min.

##### 1-(2-Acetylcyclohex-2-en-1-yl)propan-2-one (**11**)

A 30 mg portion of **S2** (0.165 mmol) gave the product as a yellow oil (21 mg, 72%). The NMR shift values are consistent with previously reported data.^18^ Enantiomeric excess was determined by HPLC on a Chiralpak AD-H column, hexane/2-propanol = 96:4, flow rate = 0.5 mL/min; 21 °C; *t*_major_ = 16.52 min, *t*_minor_ = 19.34 min.

##### 1-(2-Benzoylcyclohex-2-en-1-yl)propan-2-one (**12**)

A 40 mg portion of **S3** (0.165 mmol) gave the product as a yellow oil (17 mg, 42%). The NMR shift values are consistent with previously reported data.^18^

##### 2-(2-Benzoyl-*1H*-inden-1-yl)-1-phenylethan-1-one (**13**)

56 mg of **S4** (0.165 mmol) gave the product as a yellow oil (36 mg, 65%). The NMR shift values are consistent with previously reported data.^27^ The enantiomeric excess was determined by HPLC on a Chiralpak AD-H column, hexane/2-propanol = 96:4, flow rate = 0.5 mL/min; 21 °C; *t*_major_ = 26.67 min, *t*_minor_ = 32.54 min.

##### Benzyl 2-(2-benzoylcyclohex-2-en-1-yl)acetate (**14**)

55 mg of **S5** (0.165 mmol) gave the product as a yellow oil (41 mg, 74%). ^1^H NMR (600 MHz, CDCl_3_) δ 7.7–7.6 (m, 2H), 7.6–7.4 (m, 1H), 7.4–7.4 (m, 2H), 7.3 (m, 5H), 6.6–6.5 (m, 1H), 5.1 (s, 2H), 3.4 (m, 1H), 2.7 (dd, *J* = 15.0, 4.5 Hz, 1H), 2.5 (dd, *J* = 15.1, 9.0 Hz, 1H), 2.3–2.2 (m, 2H), 1.9–1.6 (m, 4H). ^13^C{^1^H} NMR (150 MHz, CDCl_3_) δ: 203.8, 172.1, 135.7, 134.0, 133.4, 132.2, 130.0, 129.5, 129.3, 129.1, 128.9, 128.7, 128.3, 128.0, 125.5, 125.3, 66.8, 45.5, 40.3, 39.7, 35.6. [ = −4.421 (c 0.5, MeOH). The enantiomeric excess was determined by HPLC on a Chiralpak AD-H column, hexane: 2-propanol 96:4, flow rate = 0.5 mL/min; 21 °C; *t*_major_ = 33.59 min, *t*_minor_ = 36.28 min. HRMS (ESI): calcd. for C_22_H_22_O_3_Na [M + Na]^+^ 357.1461, found 357.1461.

##### Ethyl 2-(2-benzoylcyclohex-2-en-1-yl)acetate (**15**)

45 mg of **S6** (0.165 mmol) gave the product as a yellow oil (37 mg, 82%). The NMR shift values are consistent with previously reported data.^18^ The enantiomeric excess was determined by HPLC on Chiralpak AD-H column, hexane/2-propanol 96:4, flow rate = 0.5 mL/min; 21 °C; *t*_major_ = 21.63 min, *t*_minor_ = 26.87 min.

## Supporting Information Available

^1^H and ^13^C NMR spectra, HRMS spectra, and HPLC chromatograms (PDF)
