# Supplementary material for: Catalytic Enantioselective Rauhut–Currier Reaction Mediated by Lithium Selenolates
Source: ACS Omega. 2025 Mar 18;10(12):11854–60. doi: 10.1021/acsomega.4c08290 (PMC11966293; doi:10.1021/acsomega.4c08290)
Supplement: Supplementary file 1 — ao4c08290_si_001.pdf [file ao4c08290_si_001.pdf]

Catalytic enantioselective Rauhut-Currier reaction mediated by lithium selenolates.

Gabriela Całka-Kuc[a], Seweryn Żubrowski[a], Szymon Buda*[a]
[a]Faculty of Chemistry, Jagiellonian University, Gronostajowa 2, 30-387 Krakow
[b]Jagiellonian University, Doctoral School of Exact and Natural Sciences, Łojasiewicza 11, 30-348 Kraków, Poland
E-mail: szymon.buda@uj.edu.pl

Table of Contents:

Experimental procedures	S2-S11
^{1}H NMR and ^{13}C NMR Spectra, HPLC Chromatograms, HRMS spectra	S12-S40

Experimental procedure:

Preparation of (*S*)-1,1-binaphtalene-2,2’-diol derivatives L2-L15:

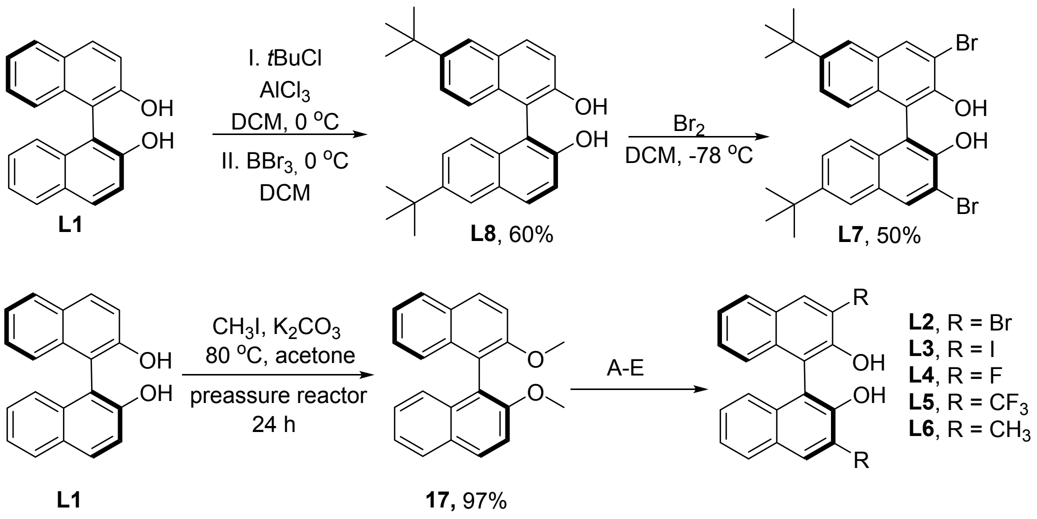

A: I. *n*BuLi, TMEDA, Br_2,, II. BBr_3, 0 °C, DCM, (**L2**, 49%)
B: I. *n*BuLi, TMEDA, I_2, -78 °C, Et_2O, (**19**, 55%) II. BBr_3, 0 °C, DCM, (**L3**, 53%)
C: I. *n*BuLi, TMEDA, NFSI, II. BBr_3, 0 °C, DCM, (**L4**, 39%)
D: I. $FSO_2CF_2CO_2Me$, CuI, DMF, (**20**, 53%) II. BBr_3, 0 °C, DCM, (**L5**, 49%)
E: I. *n*BuLi, TMEDA, CH_3I, II. BBr_3, 0 °C, DCM, (**L6**, 44%)

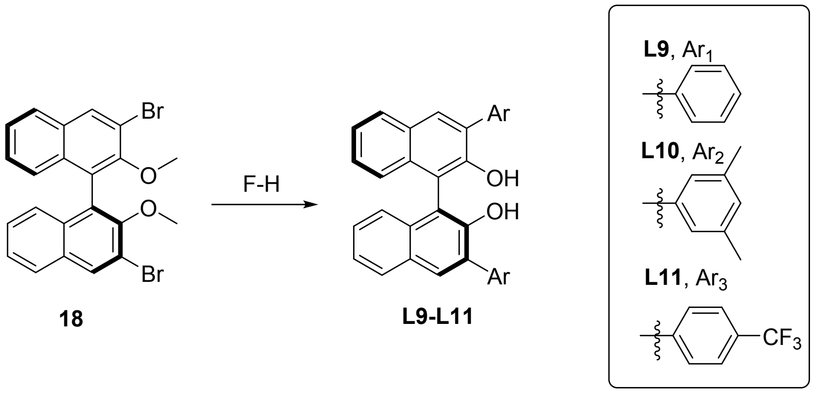

F: I $Pd(PPh_3)_4$, DME, $Ar_1B(OH)_2$ $NaHCO_3$ (aq), reflux 18 h II. BBr_3, 0 °C, DCM, (**L9**, 86%)
G: I $Pd(PPh_3)_4$, DME, $Ar_2B(OH)_2$, $NaHCO_3$ (aq) reflux, 18 h II. BBr_3, 0 °C, DCM, (**L10**, 96%)
H: I $Pd(PPh_3)_4$, DME, $Ar_3B(OH)_2$,, $NaHCO_3$ (aq), reflux 18 h, II. BBr_3, 0 °C, DCM, (**L11**, 82%)

Scheme S1. Preparation of (*S*)-1,1-binaphtalene-2,2’-diol derivatives

General procedure for the preparation of *(S)-2,2'-dimethoxy-1,1'-binaphthalene* **(17)** To a mixture of (*S*)-BINOL (1.75 mmol) and K_2CO_3 (17.5 mmol) in acetone (50 mL) was added iodomethane (17.5 mmol). The reaction was heated in a pressure reactor for 24 h. After this time, the reaction mixture was transferred to a flask and evaporated to dryness. The precipitate obtained was dissolved in 50 mL of H_2O and extracted three times with DCM (20 mL). Organic phases were combined, washed with brine, and dried over $MgSO_4$. The solution was then

evaporated to give a white crystalline solid. The NMR shift values are consistent with previously reported data.[1]

Preparation of 3,3'-disubstituted (*S*)-2,2'-dimethoxy-1,1'-binaphtalene derivatives:

(S)-3,3'-dibromo-2,2'-dimethoxy-1,1'-binaphthalene **(18)** To a 100 mL flask containing 20 mL of diethyl ether and 1.1 mL of TMEDA (6 mmol), 6 ml of *n*-BuLi (1.6 M in hexane, 9 mmol) was added and the reaction mixture was stirred for 15 min at room temperature. After this time, 1 g of (S)-2,2′-dimethoxy-1,1′-binaphthalene **17** (3 mmol) was added and the mixture was stirred constant for 24 h. After the desired time had elapsed, the mixture was cooled to -78 °C and 2 ml of bromine was carefully added dropwise (60 mmol) while continuing to stir for 4 h at room temperature. Then 50 mL of saturated $Na_2S_2O_3$ solution was added and the mixture was stirred for another 2 h. Then it was transferred to a separating funnel and extracted with diethyl ether with 20 mL portions. The combined organic fractions were washed with brine and dried over anhydrous magnesium sulfate. The product was purified by column chromatography in hexane: ethyl acetate 9: 1.Yield 77%. The NMR shift values are consistent with previously reported data.[1]

(S)-3,3'-diodo-2,2'-dimethoxy-1,1'-binaphthalene **(19)** In a 100 mL flask, 20 mL of diethyl ether and 1.1 mL of TMEDA (6 mmol) were placed. Then 6 ml of *n*-BuLi (1.6 M in hexane, 9 mmol)) was added and the mixture was stirred for 15 min at RT. Then, 1 g of (*S*)-2,2′-dimethoxy-1,1′-binaphthalene (3 mmol) was added and, under constant stirring, it was left for 24 h. After the desired time had elapsed, the mixture was cooled to -78 °C and 9.7 g of iodine (60 mmol) were added in portions, with continued stirring for 4 h at room temperature. Next, 50 mL of saturated Na_2SO_3 solution was then added and the reaction was stirred for an additional 2 h. This mixture was transferred to a separatory funnel and extracted twice with diethyl ether with 20 mL portions. The combined organic fractions were washed with brine and dried over anhydrous sulfate. The product was purified by column chromatography in hexane: ethyl acetate 9: 1. Yield 74%. The NMR shift values are consistent with previously reported data.[2]

(S)-2,2'-dimethoxy-3,3'-bis(trifluoromethyl)-1,1'-binaphthalene **(20)** To 1 g of (*S*)-3,3′-diiodo-2,2′-dimethoxy-1,1′-binaphthalene (1.8 mmol) in 2 mL of DMF was added 1 mL $FSO_2CF_2CO_2Me$ (4 eq, 7 mmol), 1 mL HMPA (4 eq, 7 mmol) and 0.09 g of CuI (2 eq, 3.6 mmol). The mixture was heated at 80 °C for 24 h. After the specified time, it was cooled to RT and 5 ml of DCM was added. The mixture was then washed with water (2 x 1 mL) and dried over anhydrous magnesium sulfate. The organic phases were combined and concentrated in an evaporator. The product was purified by column chromatography in hexane: ethyl acetate 9:1. Yield 53%. The NMR shift values are consistent with previously reported data.[3]

The above compounds were subjected to the deprotection procedure: BBr_3 (1.3 ml of 1M in DCM) was added dropwise to 5 ml of a solution of the (*S*)-2,2'-methoxybinaphthalene (2 mmol) in DCM at 0 °C. The mixture was allowed to stir at RT for 6 h. BBr_3 was neutralized by cooling the mixture to 0 °C and then adding 4 ml of water. It was extracted with DCM (6 x 10 mL), washed with brine, dried over anhydrous magnesium sulfate and evaporated to give the product in quantitative yield.

The following compounds were obtained by this procedure:
(S)-3,3'-dibromo-[1,1'-binaphthalene]-2,2'-diol **(L2)** The NMR shift values are consistent with previously reported data.[1]

(S)-3,3'-diiodo-[1,1'-binaphthalene]-2,2'-diol **(L3)** The NMR shift values are consistent with previously reported data.[2]

(S)-3,3'-bis(trifluoromethyl)-[1,1'-binaphthalene]-2,2'-diol **(L5)** The NMR shift values are consistent with previously reported data.[3]

(S)-3,3'-difluoro-[1,1'-binaphthalene]-2,2'-diol **(L4)** In a 100 mL flask, 20 mL of THF and 1.1 mL of TMEDA (6 mmol) were placed. Then 6 ml of *n*-BuLi (1.6 M in hexane, 9 mmol) was added and the mixture was stirred for 15 min at RT. After this time, 1 g of (*S*)-2,2′-dimethoxy-1,1′-binaphthalene (3 mmol) was added and, under constant stirring, it was left for 24 h. After the desired time had elapsed, the mixture was cooled to -78 °C and 2.4 g of 2.4 g were added in portions. NFSI (60 mmol), continued stirring for 4 h at room temperature. 50 mL of saturated Na_2SO_3 solution was then added and stirred for another 2 h. This was transferred to a separatory funnel and extracted twice with diethyl ether in 20 mL portions. The combined organic fractions were washed with brine and dried over anhydrous sulfate. The solvent was evaporated under reduced pressure and the resulting mixture was dissolved in 5 ml of DCM, cooled to 0 °C, and 1.3 ml of BBr_3 (1M in DCM) was added dropwise. The mixture was stirred at RT for 6 h. BBr_3 was neutralized by cooling the mixture to 0 °C and then adding 4 ml of water. It was extracted with DCM (6 x 10 mL), washed with brine, dried over anhydrous magnesium sulfate and evaporated. The product was purified by column chromatography in hexane: ethyl acetate 6: 1. Yield 36%. The NMR shift values are consistent with previously reported data.[4]

(S)-3,3'-dimethyl-[1,1'-binaphtalene]-2,2'-diol **(L6):** 1.2 ml of TMEDA (7.6 mmol) was added to a suspension of 1 g of (*S*)-2,2′-dimethoxy-1,1′-binaphthalene (3.2 mmol) in 30 mL of anhydrous Et_2O under argon. The mixture was cooled to 0 °C, *n*-BuLi 8.8 mL (1.6 M in hexane, 16.1 mmol) was added and stirring was continued for 1.5 h. The mixture was then warmed to room temperature and refluxed for another 24 h. After the specified time had elapsed, the mixture was cooled to 0 °C and 0.8 ml of CH_3I (11 mmol) was added with continued stirring at room temperature for 24 h. The reaction was quenched by adding 15 ml of water. It was then extracted with two portions of DCM (10 mL), the organic layers were combined, dried over anhydrous magnesium sulfate and the solvent was evaporated. The resulting product was dissolved in 5 ml of DCM, cooled to 0 °C, and 1.3 ml of BBr_3 (1M in DCM) was added dropwise. The mixture was stirred at RT for 6 h. BBr_3 was neutralized by cooling the mixture to 0 °C and then adding 4 ml of water. It was extracted with DCM (6 x 10 mL), washed with brine, dried over anhydrous magnesium sulfate and evaporated. The product was purified using column chromatography in hexane: ethyl acetate 6:1**.** Yield 66%. The NMR shift values are consistent with previously reported data.[4]

(S)-3,3'-diphenyl-[1,1'-binaphthalene]-2,2'-diol **(L9)** 8 ml of DME and 1 g of (*S*)-3,3′-dibromo-2,2′-dimethoxy-1,1′-binaphthalene (1.5 mmol) were introduced into a 50 mL round bottom

flask and the mixture was bubbled through. Then 0.12 g of $Pd(PPh_3)_4$ (0.1 mmol) was added and stirred for 30 min at room temperature. After this time, 0.452 g of phenylboronic acid (3.5 mmol) was introduced, followed by the addition of 10 mL of 0.1 M sodium bicarbonate solution. The resulting mixture was heated to reflux for 16 h. After this time, 10 mL of cooled ethyl acetate was added and everything was transferred to a separate funnel. The organic phase was separated, washed with brine, and then dried over anhydrous magnesium sulfate. The solvent was evaporated and the oil obtained was purified by column chromatography in hexane: ethyl acetate 10: 1. The resulting product was dissolved in 5 ml of DCM, cooled to 0 °C, and 1.3 ml of BBr_3 (1M in DCM) was added dropwise. The mixture was stirred at RT for 6 h. BBr_3 was neutralized by cooling the mixture to 0 °C and then adding 4 ml of water. It was extracted with DCM (6 x 10 mL), washed with brine, dried over anhydrous magnesium sulfate and evaporated. The product was purified by column chromatography in hexane: ethyl acetate 6: 1. Yield 92%. The NMR shift values are consistent with previously reported data.[1]

(S)-3,3'-bis(3,5-dimethylphenyl)-[1,1'-binaphthalene]-2,2'-diol **(L10)** 12 ml of DME and 0.5 g of (S)-3,3′-dibromo-2,2′-dimethoxy-1,1′-binaphthalene (0.75 mmol) were placed into a 50 mL round bottom flask and 0.06 g of $Pd(PPh_3)_4$ (0.05 mmol) was added and stirred for 30 min at room temperature. At this time, 0.775 g of 3,5-dimethylphenylboronic acid (3.5 mmol) were added, followed by the addition of 10 mL of a 1M sodium bicarbonate solution. The resulting mixture was heated to reflux for 16 h. After the allotted time, 10 mL of cooled DCM was added and the mixture was transferred to a separatory funnel. The organic phase was separated, washed with brine and then dried over anhydrous magnesium sulfate. The solvent was evaporated and the resulting oil was dissolved in 5 ml of DCM, cooled to 0 °C, and 1.3 ml of BBr_3 (1M in DCM) was added dropwise. The mixture was stirred at RT for 6 h. BBr_3 was neutralized by cooling the mixture to 0 °C and then adding 4 ml of water. It was extracted with DCM (6 x 10 mL), washed with brine, dried over anhydrous magnesium sulfate and evaporated. The product was purified by column chromatography in hexane: ethyl acetate 5: 1. Yield 88%. The NMR shift values are consistent with previously reported data.[5]

(S)-3,3'-bis(4-(trifluoromethyl)phenyl)-1,1'-binaphthalene-2,2'-diol **(L11)** 12 ml of DME and 0.5 g of (*S*)-3,3′-dibromo-2,2′-dimethoxy-1,1′-binaphthalene (0.75 mmol) were introduced into a 50 mL round bottom flask and the mixture was bubbled through. Then 0.06 g of $Pd(PPh_3)_4$ (0.05 mmol) was added and stirred for 30 min at room temperature. At this time, 0.72 g of 3,5-dimethylphenylboronic acid (3.5 mmol) was introduced, followed by the addition of 10 mL of a 1M sodium bicarbonate solution. The resulting mixture was heated to reflux for 16 h. After the allotted time, 10 mL of cooled DCM was added and the whole was transferred to a separatory funnel. The organic phase was separated, washed with brine, and then dried over anhydrous magnesium sulfate. The solvent was evaporated, and the resulting oil was dissolved in 5 ml of DCM, cooled to 0 °C, and 1.3 ml of BBr_3 (1M in DCM) was added dropwise. The mixture was allowed to stir at RT for 6 h. BBr_3 was neutralized by cooling the mixture to 0 °C and then adding 4 ml of water. It was extracted with DCM (6 x 10 mL), washed with brine, dried over anhydrous magnesium sulfate and evaporated. The product was purified using column chromatography in hexane: ethyl acetate 5:1. Yield 84%. The NMR shift values are consistent with previously reported data.[5]

(S)-6,6'-di-tertbutyl-[1,1'-binaphthalene]-2,2'-diol **(L8)** A solution of (*S*)-BINOL (1 g, 2.5 mmol) in anhydrous DCM was cooled to -78 °C and 4.5 ml of *tert*-butyl chloride (0.38 g, 40 mmol) was added, followed by 0.53 g of anhydrous $AlCl_3$ while continuing stirring for 12 h. The reaction was quenched by adding 10 mL of cold water and extraction was performed with three 20 mL portions of cooled DCM. The organic layers were washed with a saturated sodium bicarbonate solution then dried over anhydrous sodium sulfate and the solvent was evaporated. Finally, 30 mL of hexane was added to remove the insoluble impurities that were filtered out. The solvent was slowly evaporated at 25 °C to give a white crystalline solid with 60% yield. The NMR shift values are consistent with previously reported data.[6]

(S)-3,3'-dibromo-6,6'-di-tert-butyl-[1,1'-binaphthalene]-2,2'-diol **(17)** A solution of 1 g of (*S*)-6,6'-di-tert-butyl-1,1'-binaphthalene-2,2'-diol (2.5 mmol) in dichloromethane (30 mL) was cooled to -78 °C. After 15 min, bromine (1.1 g, 6.8 mmol in 10 mL of DCM) was added dropwise and stirred for 5 h. The mixture was then warmed to room temperature and quenched with 20 mL of sodium thiosulfate while stirring continued for 2 h. The mixture was then extracted with dichloromethane, in three 20 mL portions. The organic layers were combined, dried over anhydrous sodium sulfate (VI), and the solvent was evaporated to give a product which was then crystallized from hexane-ethyl acetate (2: 1) to give a brown solid with a 50% yield. NMR shift values are consistent with previously reported data.[6]

(S)-6,6'-dibromo-[1,1'-binaphthalene]-2,2'-diol **(15)** In a 50 mL round bottom flask, 0.86 g of (*S*)-1,1′-binaphthalene (3 mmol) was dissolved in 10 mL of dichloromethane and the mixture was cooled to -78 °C. After 30 min, 1 mL of bromine (30 mmol) was carefully added dropwise, while stirring continued for 6 h. Then 50 mL of saturated $Na_2S_2O_3$ solution was added and stirring was carried out for another 2 h at room temperature. It was then transferred to a separating funnel and extracted twice with diethyl ether in 20 mL portions. The combined organic fractions were washed with brine and dried over anhydrous magnesium sulfate. The product was purified by column chromatography in hexane: ethyl acetate 9: 1. Yellow solid, 48% yield. The NMR shift values are consistent with previously reported data.[7]

Preparation of substrate 1 and S1-S7

General procedure for the preparation of symmetrical bisenones: 2.5 eq of the ylide was dissolved in ethanol (10 mL/g of the ylide) at room temperature. 1 eq of glutaraldehyde was added, and the reaction was allowed to stir for 24 h. The solvent was then evaporated and the resulting suspension was washed several times with a mixture of 3:7 hexane and ether to precipitate triphenylphosphine oxide. The precipitate was filtered off under reduced pressure, and the resulting solution was evaporated. The resulting oil was purified by column chromatography using a mixture of hexane: ethyl acetate (9:1).

(2E,7E)-1,9-diphenyl-2,7-nonadiene-1,9-dione **(1)**

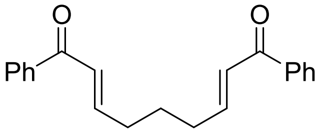

1

2 g of glutaric aldehyde (20 mmol) and 19 g 1-phenyl-2-(triphenylphosphanylidene)-ethanone (50 mmol) gave the product (**1**) as a yellow oil (4.13 g, 13.5 mmol, 68%). The NMR shift values are consistent with previously reported data.[8]

(2E,6E)-1,8-diphenylocta-2,6-diene-1,8-dione **(S1)**

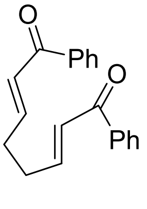

S1

1.08 g of 1,5-cyclooctadiene (10 mmol) was dissolved in 30 mL anhydrous dichloromethane and cooled to -78 °C. Ozone was bubbled through the mixture until a blue color appeared. The reaction was then flushed with argon and 2.62 g (10 mmol) of triphenylphosphine was added in one portion. The resulting mixture was allowed to warm to 0 °C, and 7.6 g (20 mmol) of 1-phenyl-2-(triphenyl-5-phosphaneylidene)ethan-1-one was added. The reaction mixture was stirred for 12 h at room temperature. The solvent was then evaporated and the resulting suspension was washed three times with a 3:7 mixture of hexane and ether. The precipitate was filtered under reduced pressure and the resulting solution was evaporated. The resulting oil was purified by column chromatography using a mixture of hexane: ethyl acetate (9:1) to give a yellow oil (1.35 g, 45%). The NMR shift values are consistent with previously reported data.[8]

(3E,8E)-undeca-3,8-diene-2,10-dione **(S2)**

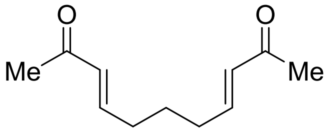

S2

2 g of glutaric aldehyde (20 mmol) and 15.9 g 1-(triphenylphosphaneylidene)propan-2-one (50 mmol) gave the product (**S2**) as an yellow oil (1.98 g, 10.5 mmol, 55%). The NMR shift values are consistent with previously reported data.[8]

(2E,2'E)-3,3'-(1,2-phenylene)bis(1-phenylprop-2-en-1-one) **(S4)**

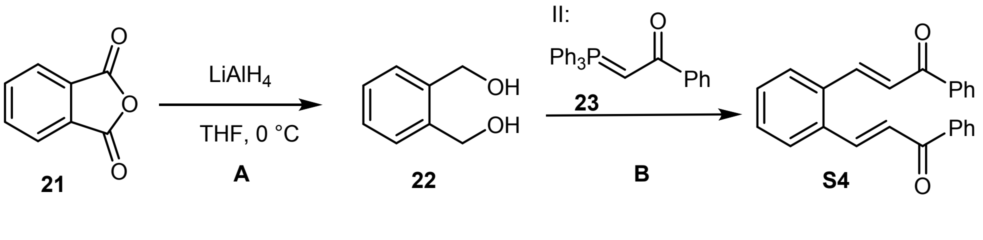

Scheme S2. Preparation of the substrate **S4**

(A) A suspension of 38 mg (1 mmol) of $LiAlH_4$ in 20 ml of anhydrous THF was cooled to 0 °C. Then 59 mg (0.4 mmol) of phthalic anhydride (**21**) was added portion wise and the mixture was allowed to warm to room temperature. In the end, it was cooled back to 0 °C and 5 ml of water: a THF mixture (1: 1) was added followed by 5 ml of methanol. After 30 min, the precipitated solid was filtered through a pad of celite and dissolved in DCM. The solution was washed with brine (10 ml) followed by water (10 ml). The organic phase was dried over anhydrous sodium sulfate, filtered, and evaporated to give a with solid (47 mg, 85%). The NMR shift values are consistent with previously reported data.[9]

(B) In 10 ml of anhydrous DCM, 28 mg (2 mmol) of **22** was dissolved and 150 mg (4 mmol) of PDC was added in portions while stirring the resulting solution for 1 h at room temperature. Then 152 mg (4 mmol) of **23** was added in one portion with continued stirring for another 24 hours. The mixture was filtered through a pad of celite and then rinsed with DCM. The solution was transferred to a separatory funnel, washed with 10 mL of brine, then with 10 mL of water. The organic layer was dried over anhydrous sodium sulfate, the solvent was evaporated, and the resulting oil was purified by column chromatography using hexane: ethyl acetate 6:1 to give a white solid (32 mg, 46%). The NMR shift values are consistent with previously reported data.[10]

General procedure for the preparation of non-symmetrical substrates:

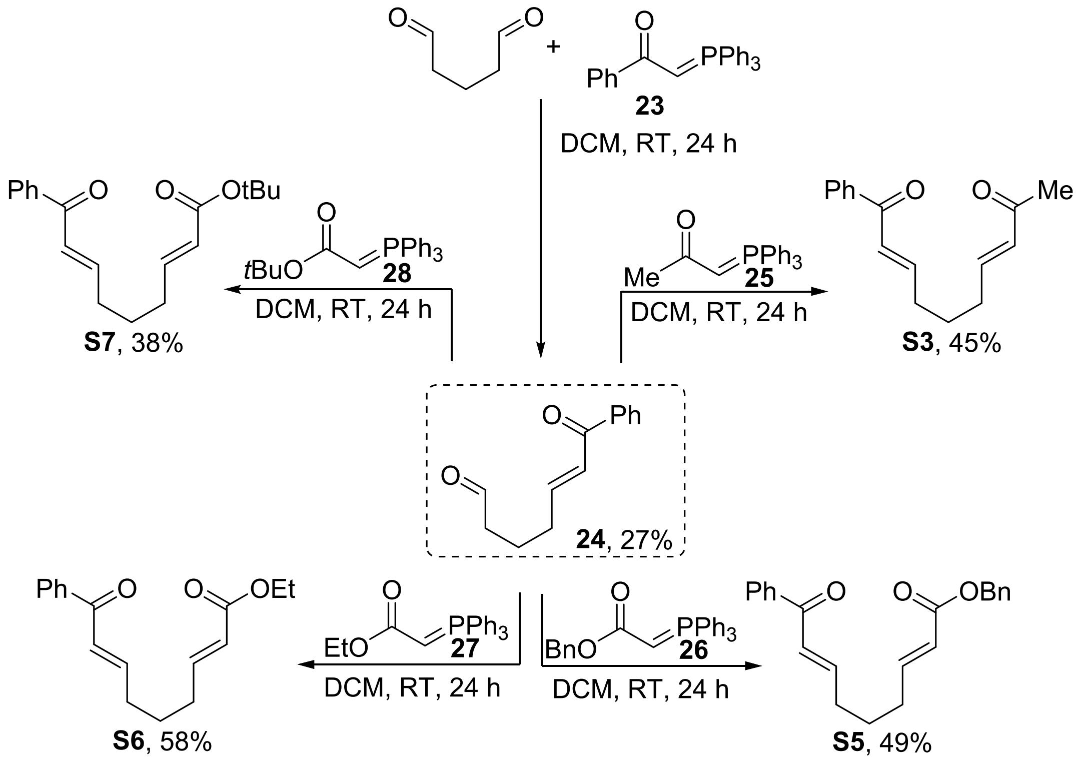

Scheme S3. Preparation of non-symmetrical substrates

(E)-7-oxo-7-phenylhept-5-enal **(24):**

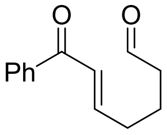

24

Ylide **23** (19 g, 50 mmol) was dissolved in 190 mL DCM at room temperature. Glutaraldehyde (2 g, 20 mmol) dissolved in 100 mL of DCM was added and the mixture was allowed to stir for 24 h. Then 300 ml of water was added and the mixture was extracted with three portions of diethyl ether (200 mL). The organic layers were evaporated and the resulting suspension was washed with a 3:7 mixture of diethyl ether and hexane. The procedure was repeated several times to remove residual triphenylphosphine oxide. The resulting oil was purified by column chromatography using hexane: ethyl acetate (9:1) to give a yellow oil (1.16 g, 5.9 mmol, 27%). The NMR shift values are consistent with previously reported data.[11]

1 eq of the ylide was dissolved in ethanol (10 mL/g of the ylide) at room temperature. Then 1 eq of **24** in 50 mL of DCM was added dropwise and the mixture was stirred for 24 h. The solvent was then evaporated and the resulting suspension was washed three times with a 3:7 mixture of hexane and ether to precipitate triphenylphosphine oxide. The precipitate was filtered under reduced pressure, and the resulting solution was evaporated. The resulting oil was purified by column chromatography using a mixture of hexane: ethyl acetate (9:1).

(2E,7E)-1-phenyldeca-2,7-diene-1,9-dione **(S3)**

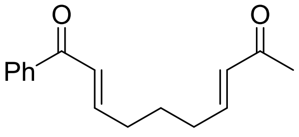

S3

A mixture of **24** (1g, 10 mmol) and 2.42 g **25** (25 mmol) in DCM gave the product (**S3**) as a colorless oil (1.09 g, 4.5 mmol, 45%). The NMR shift values are consistent with previously reported data.[8]

Benzyl (2E,7E)-9-oxo-9-phenylnona-2,7-dienoate **(S5)**

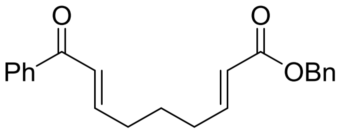

S5

A mixture of **24** (1g, 10 mmol) and 4.5 g **26** (25 mmol) in DCM gave the product (**S5**) as a colorless oil (1.64 g, 4.9 mmol, 49%).

^{1}H NMR (300 MHz, $CDCl_3$) δ 7.93 (ddd, *J* = 8.35, 1.45, 0.50 Hz, 2H), 7.58 – 7.48 (m, 1H), 7.47 – 7.39 (m, 2H), 7.39 – 7.28 (m, 5H), 7.09 – 6.98 (m, 2H), 6.97 – 6.84 (m, 1H), 5.90 (dt, *J*

= 15.65, 1.57 Hz, 1H), 5.18 (s, 2H), 2.38 – 2.17 (m, 4H), 1.66 (p, J = 7.57 Hz, 2H). $^{13}C\{^1H\}$ NMR (300 MHz, $CDCl_3$) δ 207.8, 191.2, 167.1, 149.2, 148.6, 138.4, 133.3, 129.2, 129.1, 127.0, 122.6, 60.8, 32.7, 32.1, 27.1, 14.9.
HRMS (ESI): calcd. for $C_{22}H_{22}O_3Na$ $[M + Na]^+$ 357.1461, found 357.1460.

Ethyl (2E,7E)-9-oxo-9-phenylnona-2,7-dienoate **(S6)**

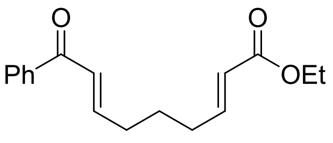

S6

A mixture of **24** (1g, 10 mmol) and 3.48 g **27** (25 mmol) in DCM gave the product (**S6**) as a colorless oil (1.58 g, 5.8 mmol, 58%). The NMR shift values are consistent with previously reported data.[9]

Tert-butyl (2E,7E)-9-oxo-9-phenylnona-2,7-dienoate **(S7)**

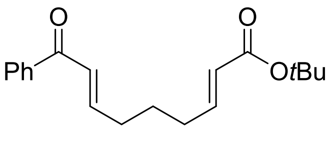

S7

A mixture of **24** (1g, 10 mmol) and 3.76 g **28** (25 mmol) in DCM gave the product (**S7**) as a colorless oil (1.14 g, 3.8 mmol, 38%).
^1H NMR (300 MHz, $CDCl_3$) δ 7.98 – 7.87 (m, 2H), 7.55 (dddd, J = 8.3, 4.2, 2.1, 1.3 Hz, 2H), 7.50 – 7.42 (m, 1H), 7.42 – 7.20 (m, 1H), 7.10 – 6.95 (m, 1H), 6.96 – 6.77 (m, 1H), 5.86 – 5.71 (m, 1H), 2.45 – 2.30 (m, 2H), 2.30 – 2.13 (m, 4H), 1.84 – 1.63 (m, 9H). $^{13}C\{^1H\}$ NMR (300 MHz, $CDCl_3$) δ 190.9, 165.8, 149.2, 147.6, 138.0, 132.6, 128.6, 128.5, 126.2, 122.3, 80.2, 32.4, 28.3, 28.2, 27.6.
HRMS (ESI): calcd. for $C_{19}H_{24}O_3Na$ $[M + Na]^+$ 323.1618, found 323.1615.

General procedure for the Rauhut-Currier reaction

13 mg of selenium (0.165 mmol) was placed into the vial and 1 mL of dry and degassed THF was added. The mixture was cooled at 0 °C for 5 min, then 0.1 mL of *n*-BuLi (0.165 mmol, 1.6 M solution in hexane) was slowly added dropwise until the solution was discolored. Stirring was continued for another 5 min and then 2 eq of water and 10% of the BINOL in 0.5 mL of THF was added dropwise. The reaction was then carried out under the specified temperature conditions. After about 5 min, the appropriate starting material was added dropwise in 0.5 mL of THF by syringe pump in a flow 0.5 ml/min. After the specified time, 1 mL of H_2O_2 was added, and then the mixture was allowed to warm to room temperature. After about 15 min, extraction with ethyl acetate was performed. The organic layers were collected and dried over anhydrous $MgSO_4$. The solvent was evaporated and the resulting oil was purified by column chromatography (hexane:ethyl acetate 6:1).

Reference:

[1] Arnold, L. A.; Imbos, R.; Mandoli, A.; de Vries, A. H. M.; Naasz, R.; Feringa, B. L. Enantioselective Catalytic Conjugate Addition of Dialkylzinc Reagents Using Copper–Phosphoramidite Complexes: Ligand Variation and Non-Linear Effects. *Tetrahedron* **2000**, *18*, 2865–2878. https://doi.org/10.1016/S0040-4020(00)00142-3

[2] Brenet, S.; Baptiste, B.; Philouze, C.; Berthiol, F.; Einhorn, J. BINOL-Fused Maleimides—A New Class of C2-Symmetric Chiral Imides. *Eur. J. Org. Chem.* **2013**, *2013* (6), 1041–1045. https://doi.org/10.1002/ejoc.201201525.

[3] Chen, H.; Chen, Y.; Tang, X.; Liu, S.; Wang, R.; Hu, T.; Gao, L.; Song, Z. Rhodium-Catalyzed Reaction of Silacyclobutanes with Unactivated Alkynes to Afford Silacyclohexenes. *Angew. Chem. Int. Ed.* **2019**, *58* (14), 4743–4747. https://doi.org/10.1002/ange.201814143.

[4] Han, Y. Q.; Ding, Y.; Zhou, T.; Yan, S. Y.; Song, H.; Shi, B. F. Pd(II)-Catalyzed Enantioselective Alkynylation of Unbiased Methylene C(Sp3)–H Bonds Using 3,3′-Fluorinated-BINOL as a Chiral Ligand. *J. Am. Chem. Soc.* **2019**, *141* (11), 4558–4563. https://doi.org/10.1021/jacs.9b01124.

[5] Ahmed, I.; Clark, D. A. Rapid Synthesis of 3,3′-Bis-Arylated BINOL Derivatives Using a C–H Borylation in Situ Suzuki-Miyaura Coupling Sequence. *Org. Lett.* **2014**, *16* (16), 4332–4335. https://doi.org/10.1021/ol502126r.

[6] Balaraman, E.; Kumara Swamy, K. C. A Convenient Chromatography-Free Access to Enantiopure 6,6′-Di-Tert-Butyl-1,1′-Binaphthalene-2,2′-Diol and Its 3,3′-Dibromo, Di-Tert-Butyl and Phosphorus Derivatives: Utility in Asymmetric Synthesis. *Tetrahedron: Asymmetry* **2007**, *18* (17), 2037–2048. https://doi.org/10.1016/j.tetasy.2007.06.028.

[7] Bunzen, J.; Bruhn, T.; Bringmann, G.; Lützen, A. Synthesis and Helicate Formation of a New Family of BINOL-Based Bis(Bipyridine) Ligands. *J. Am. Chem. Soc.* **2009**, *131* (10), 3621–3630. https://doi.org/10.1021/ja807780j.

[8] Aroyan, C. E.; Dermenci, A.; Miller, S. J. Development of a Cysteine-Catalyzed Enantioselective Rauhut-Currier Reaction. *J. Org. Chem.* **2010**, *75* (17), 5784–5796. https://doi.org/10.1021/jo101018t.

[9] Dow, M.; Archetti, F.; Abrahams, K. A.; Vaz, L.; Esra, G. B.; Warriner, S.; Nelson, A. Modular Synthesis of Diverse Natural Product-Like Macrocycles: Discovery of Hits with Antimycobacterial Activity. *Chem. Eur. J.* **2017**, *23*, 7207–7211. https://doi.org/10.1002/chem.201701150.

[10] Brown, P. M.; Käppel, N.; Murphy, P. J. Tandem Michael/Michael Reactions Mediated by Phosphines or Aryl Thiolates. *Tetrahedron Lett.* **2002**, *43*, 8707–8710. https://doi.org/10.1016/j.tet.2006.11.064

[11] Medici, F.; Resta, S.; Presenti, P.; Caruso, L.; Puglisi, A.; Raimondi, L.; Rossi, S.; Benaglia, M. Stereoselective Visible-Light Catalyzed Cyclization of Bis(Enones): A Viable Approach to the Synthesis of Enantiomerically Enriched Cyclopentane Rings. *Eur. J. Org. Chem.* **2021**, *2021* (32), 4521–4524. https://doi.org/10.1002/ejoc.202100397.

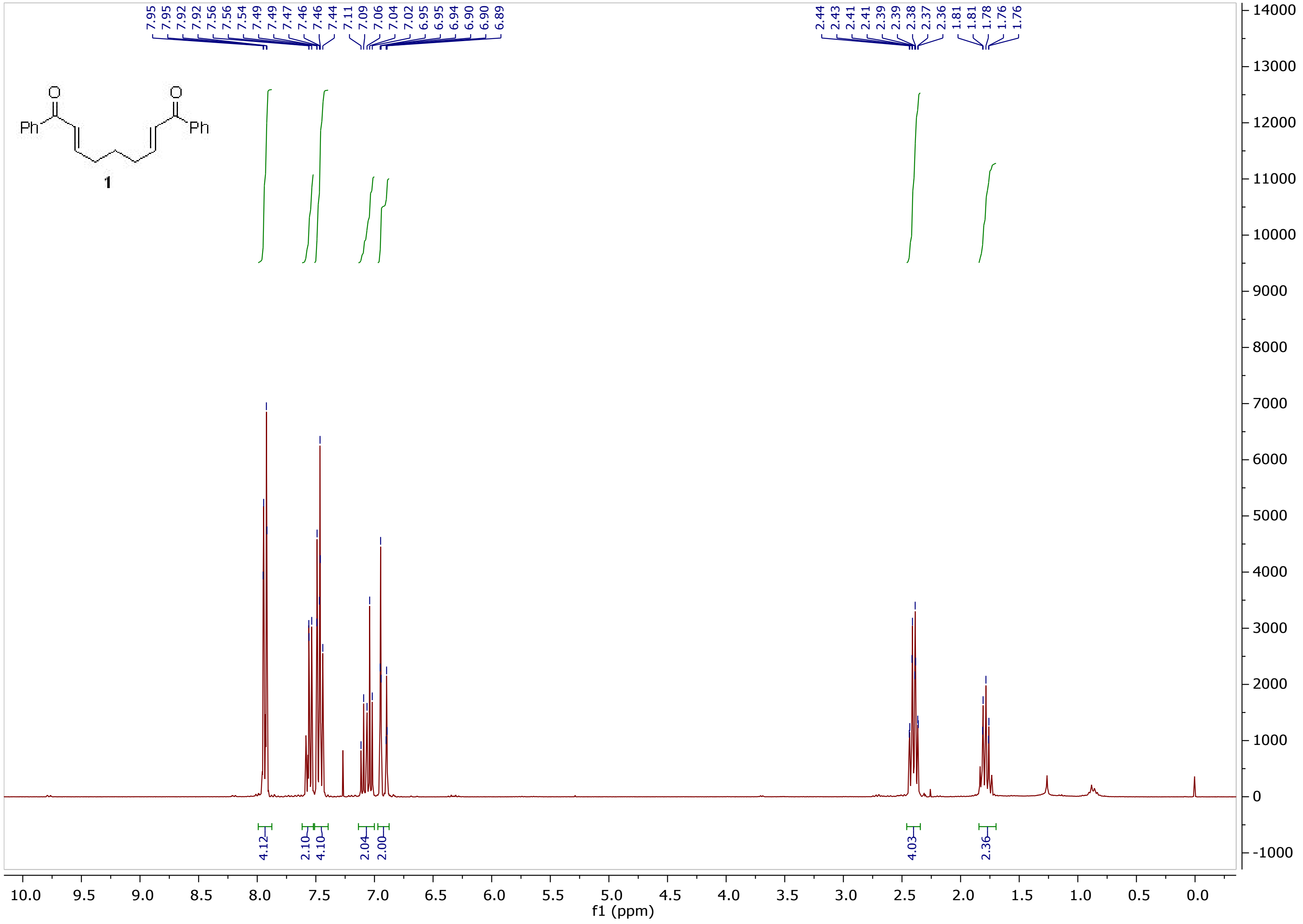

Figure 1. ^{1}H NMR for compound **1** (300 MHz, $CDCl_3$)

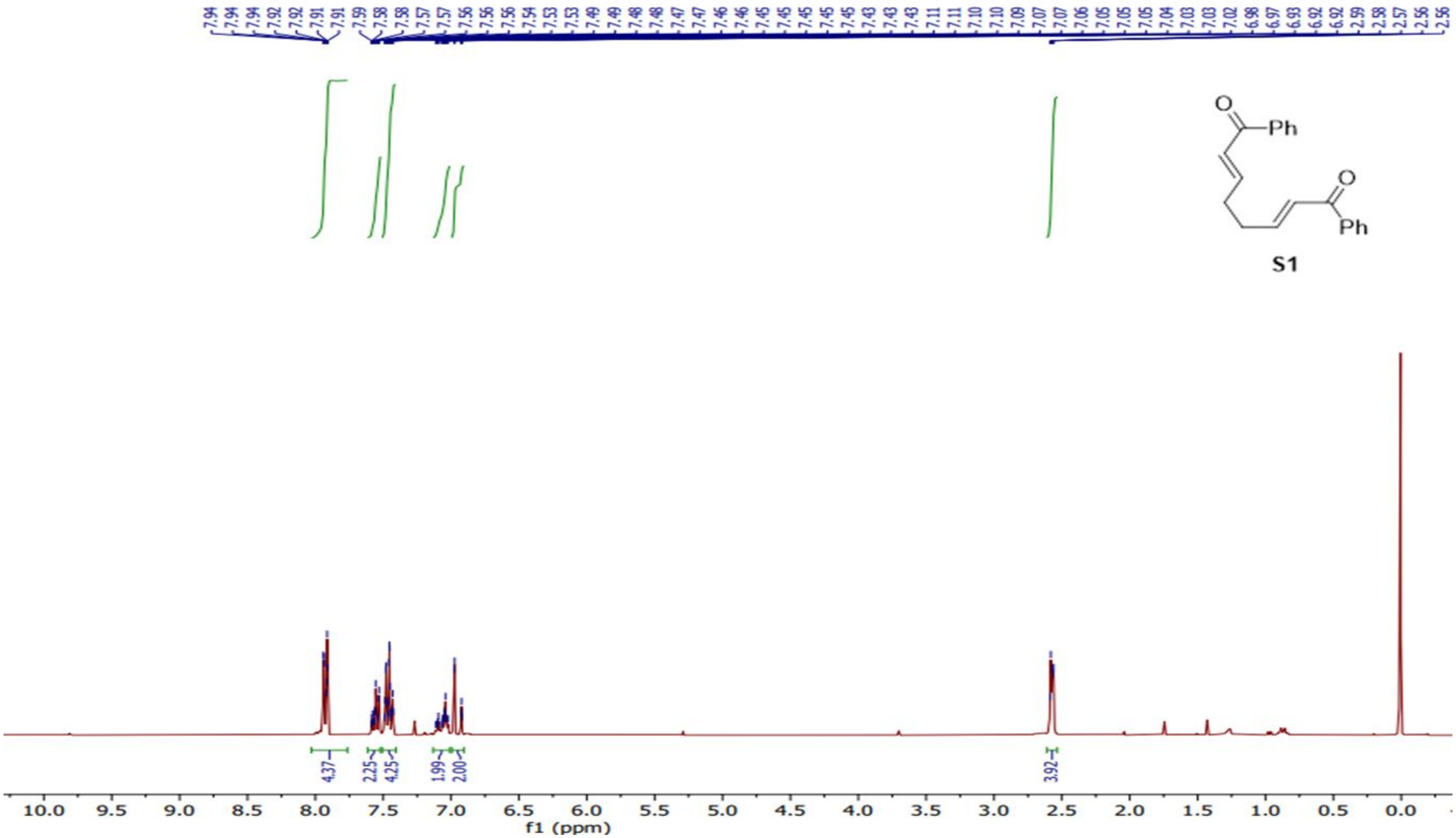

Figure 2. ^{1}H NMR for compound **S1** (300 MHz, $CDCl_3$)

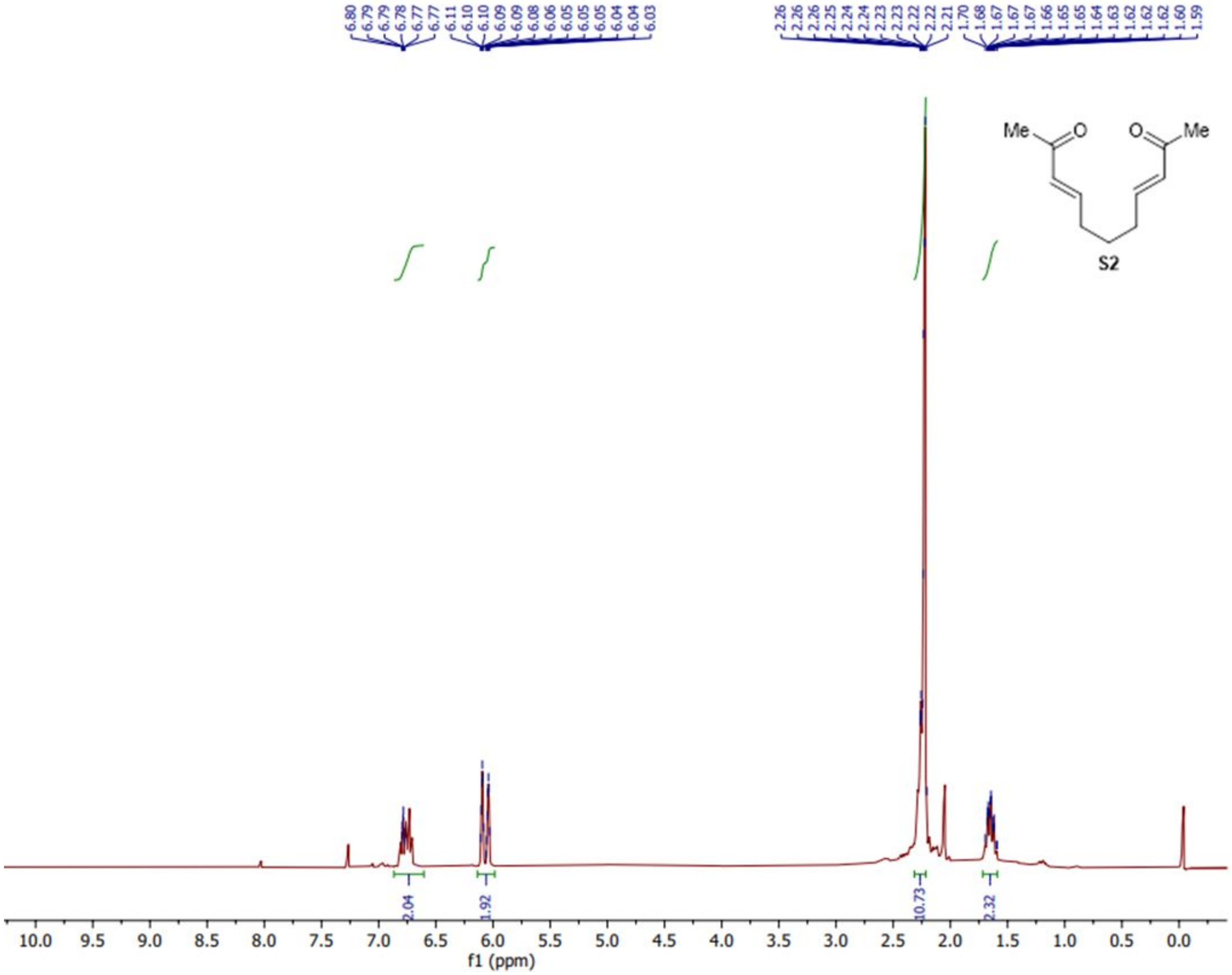

Figure 3. ^{1}H NMR for compound **S2** (300 MHz, $CDCl_3$)

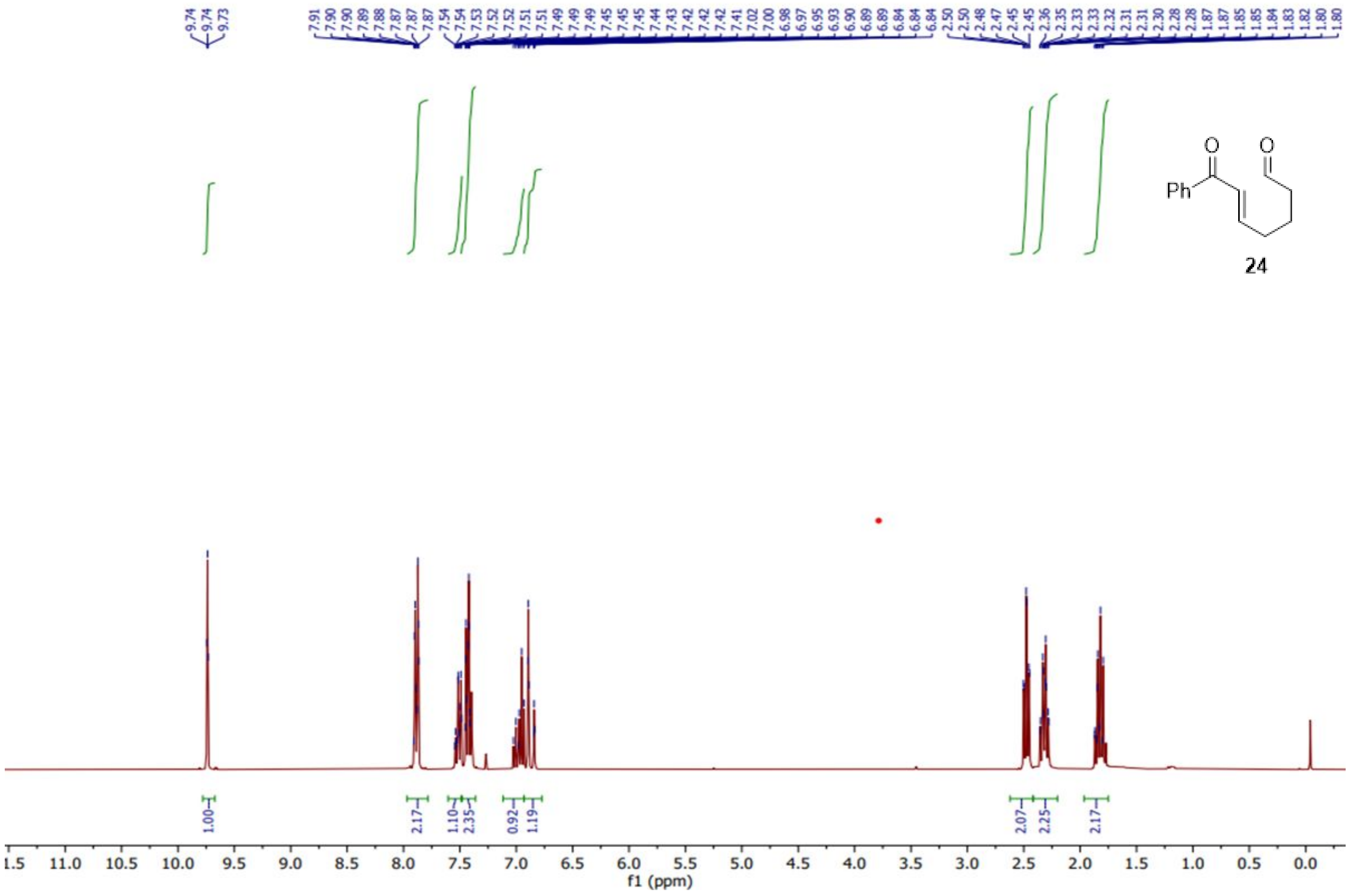

Figure 4. ^{1}H NMR for compound **24** (300 MHz, $CDCl_3$)

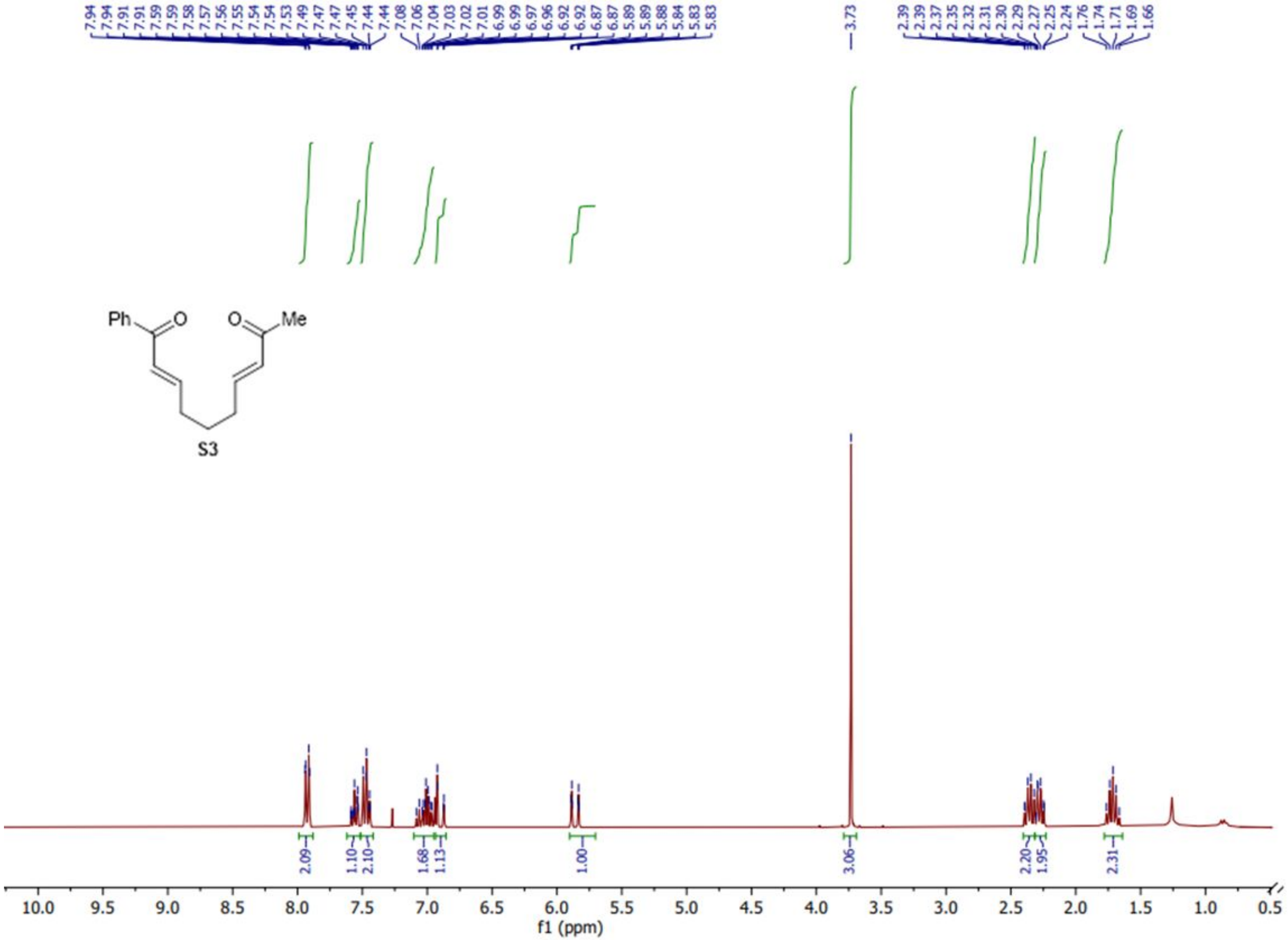

Figure 5. ^{1}H NMR for compound **S3** (300 MHz, $CDCl_3$)

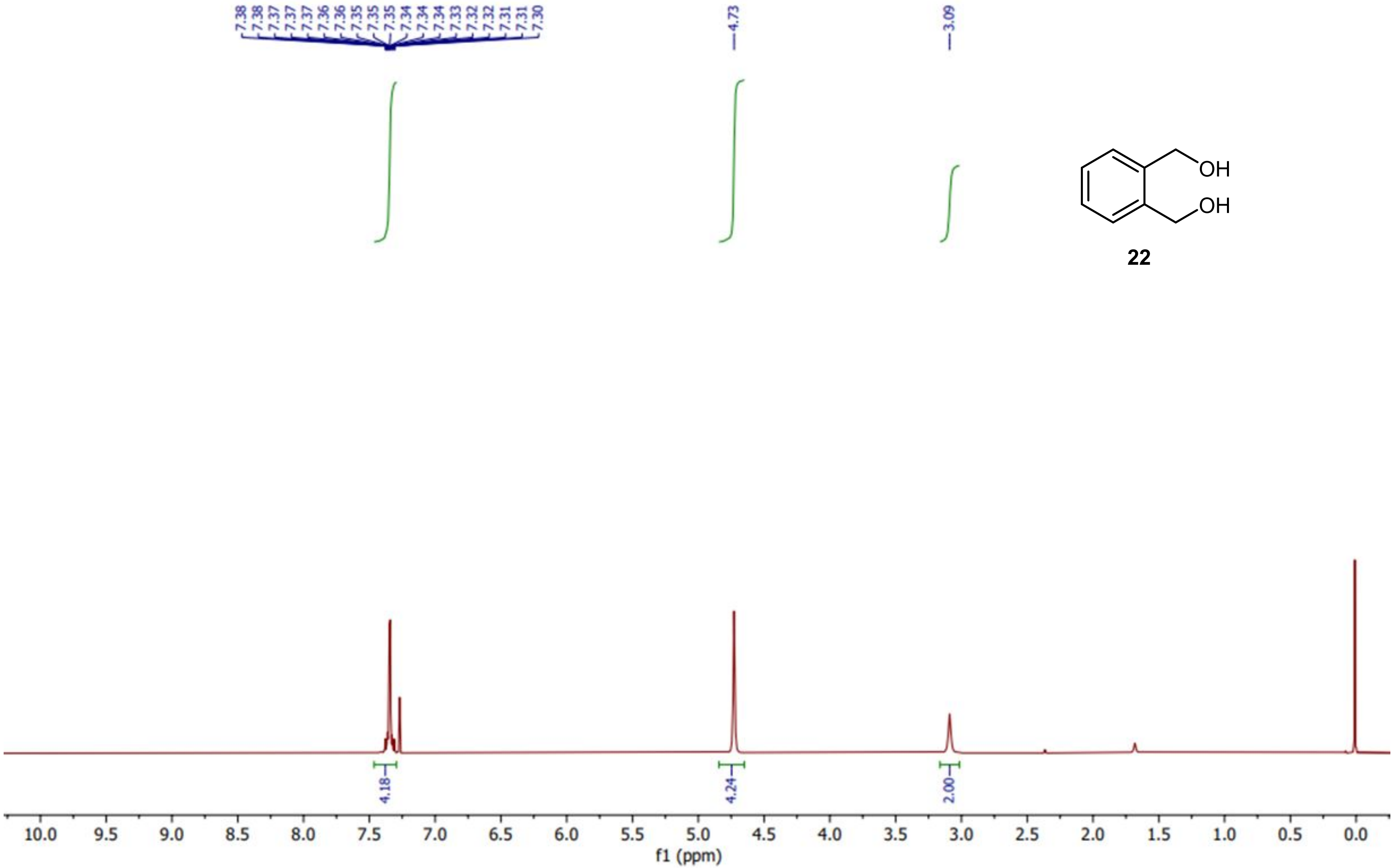

22

Figure 6. ^{1}H NMR for compound **33** (300 MHz, $CDCl_3$)

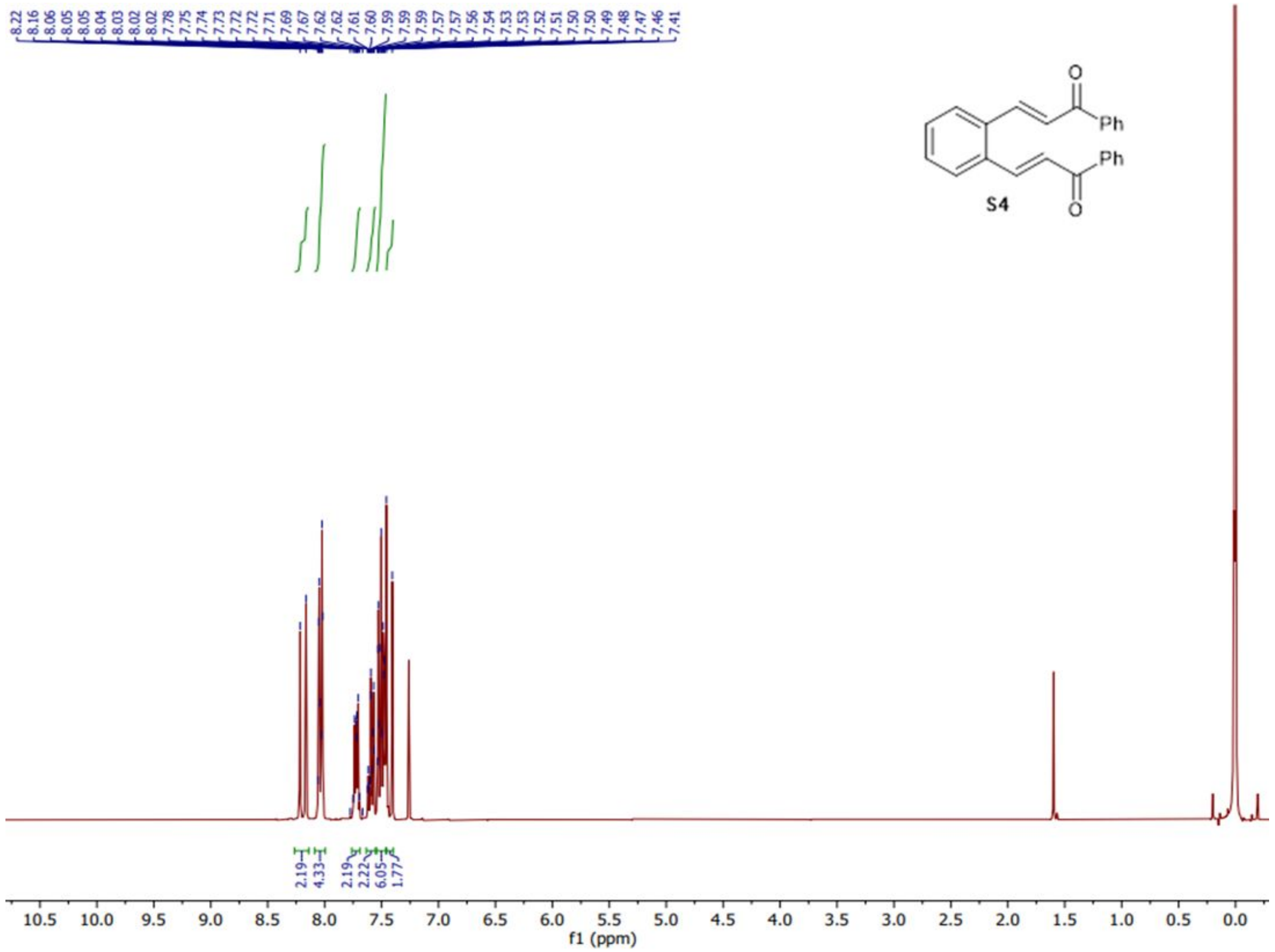

Figure 7. ^{1}H NMR for compound **S4** (300 MHz, $CDCl_3$)

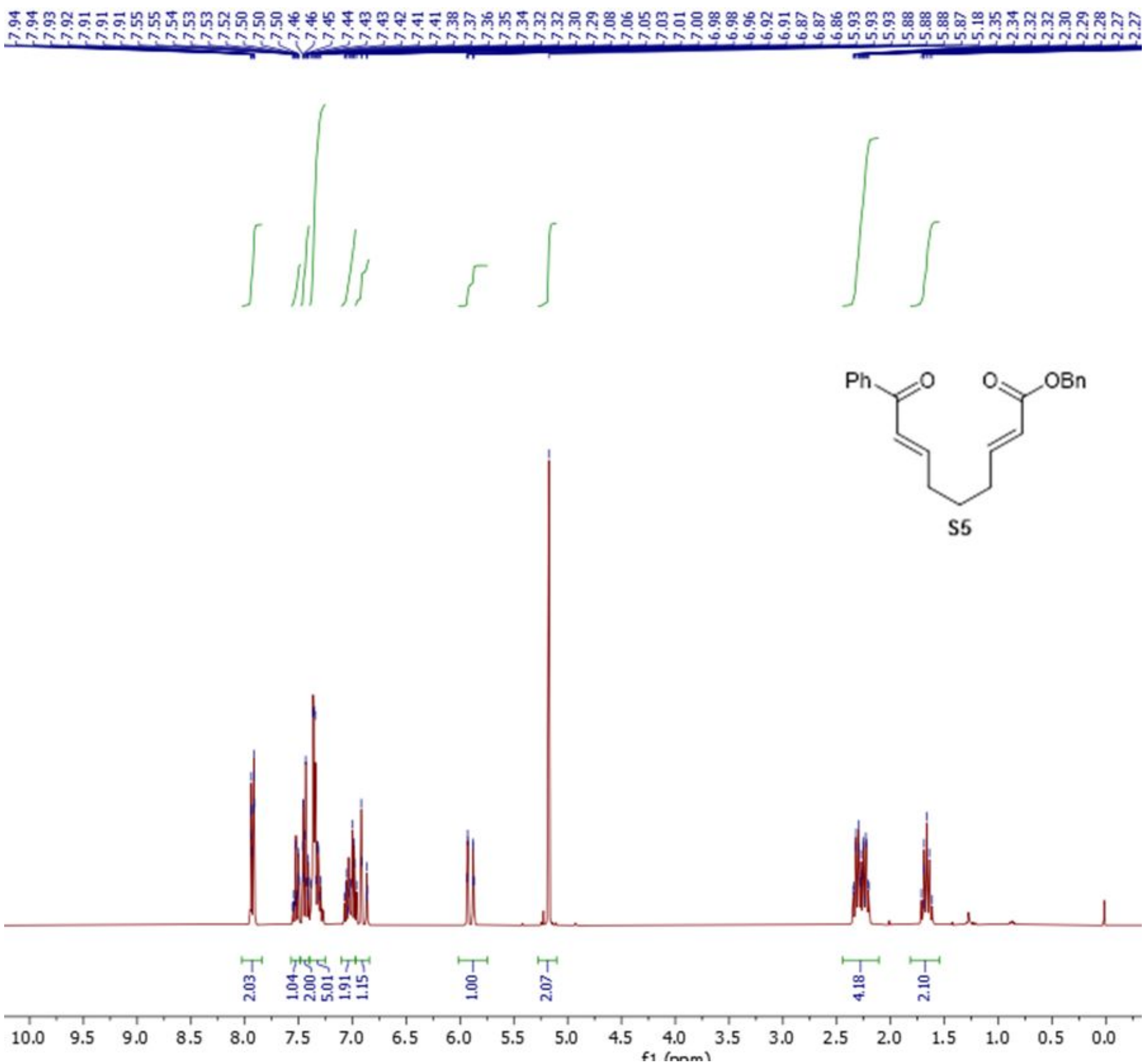

Figure 8. ^{1}H NMR for compound **S5** (300 MHz, $CDCl_3$)

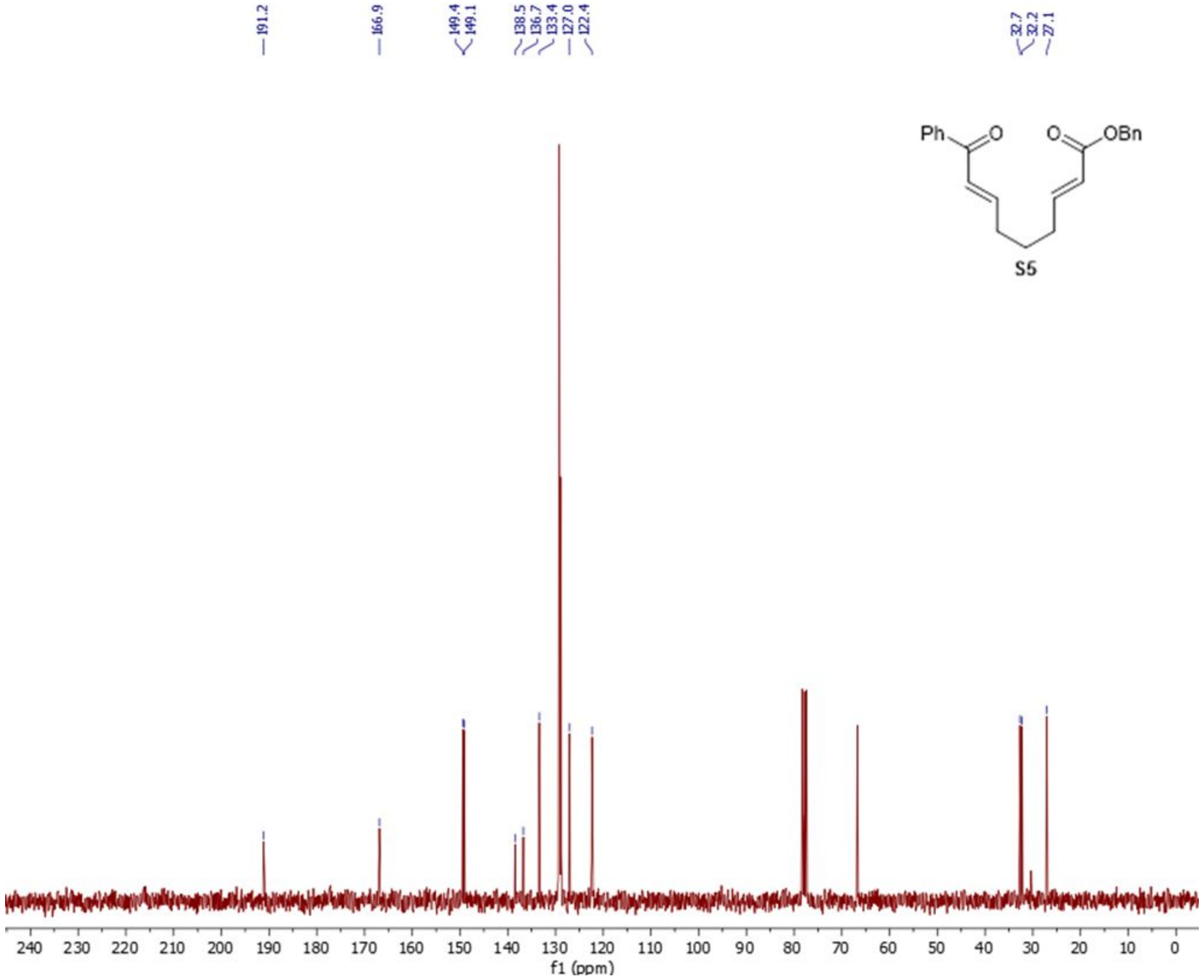

Figure 9. $^{13}C\{^{1}H\}$ NMR for compound **S5** (300 MHz, $CDCl_3$)

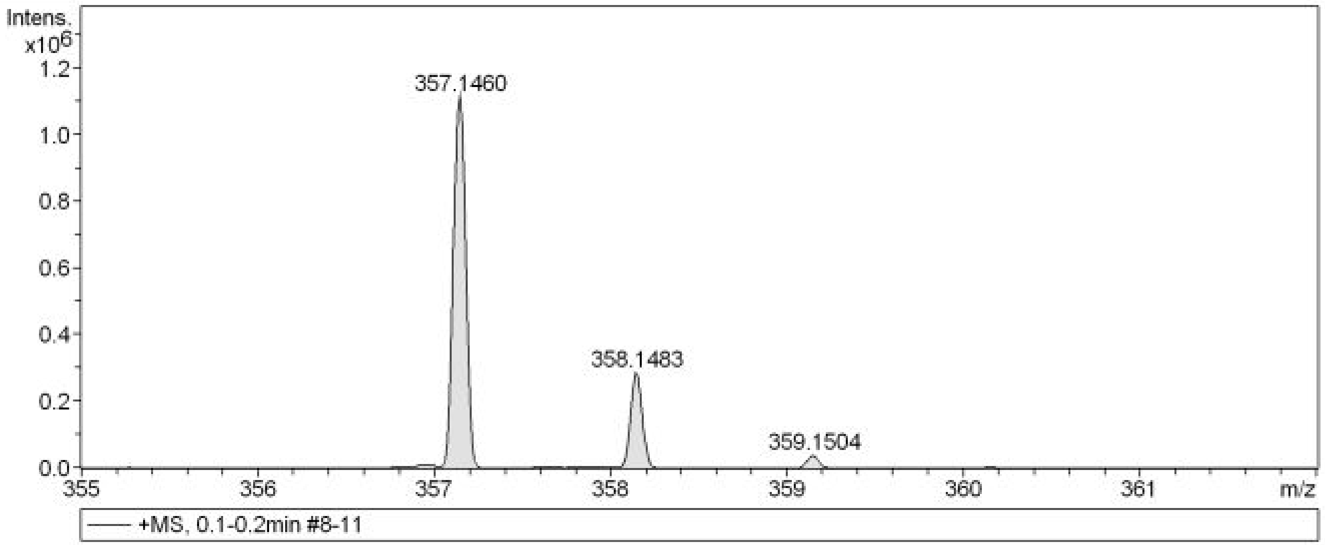

Figure 10. HRMS spectra for compound **S5**

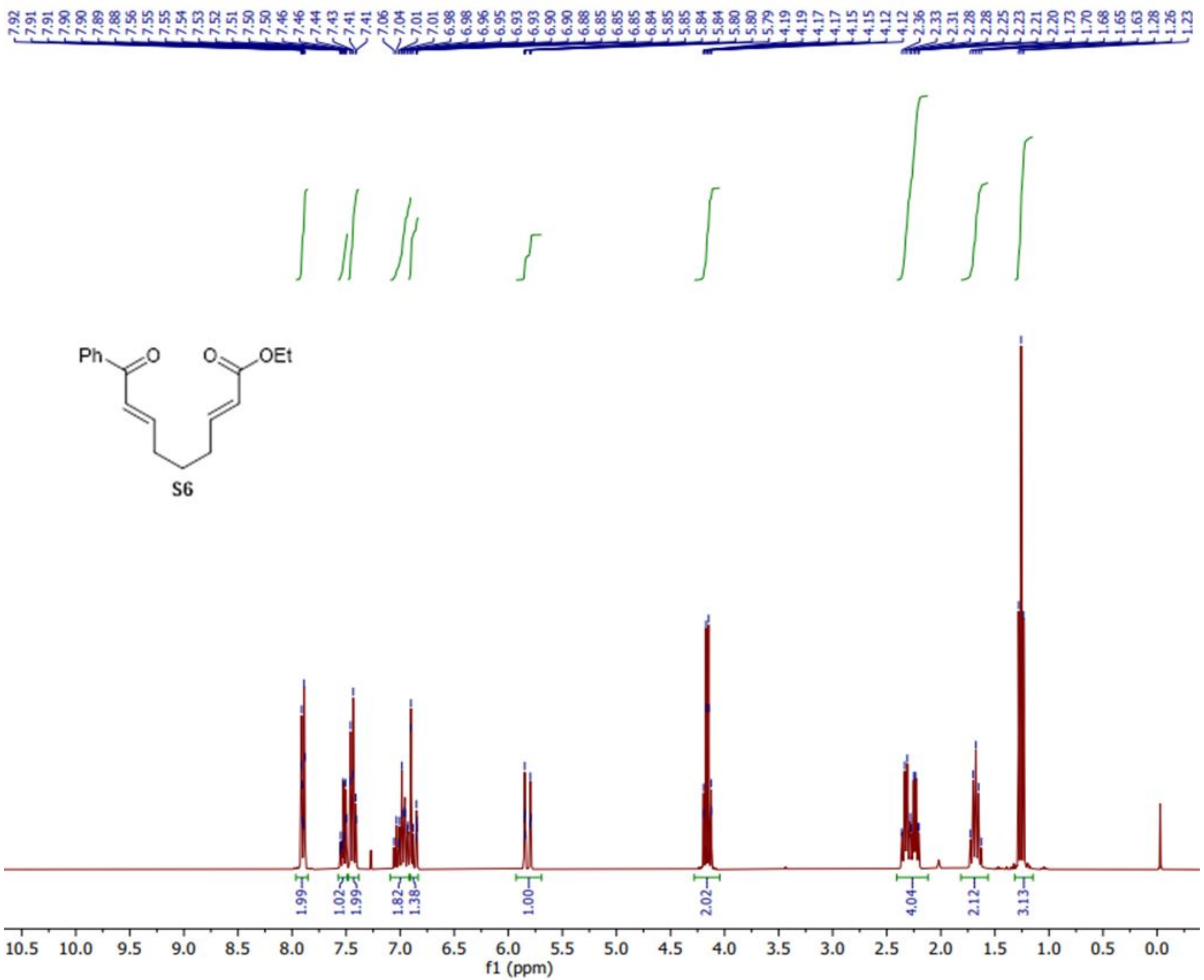

Figure 11. ^{1}H NMR for compound **S6** (300 MHz, $CDCl_3$)

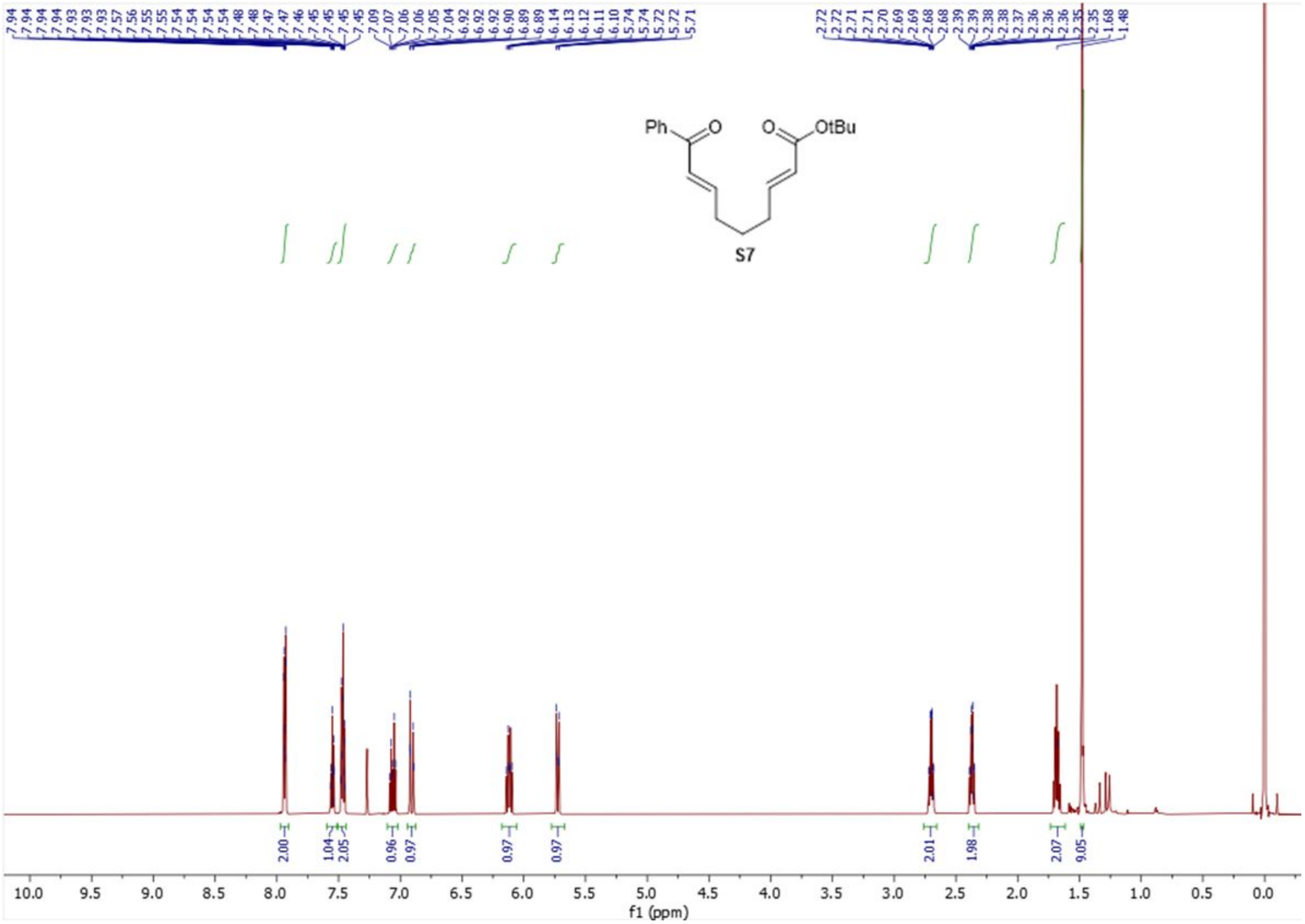

Figure 12. ^{1}H NMR for compound **S7** (600 MHz, $CDCl_3$)

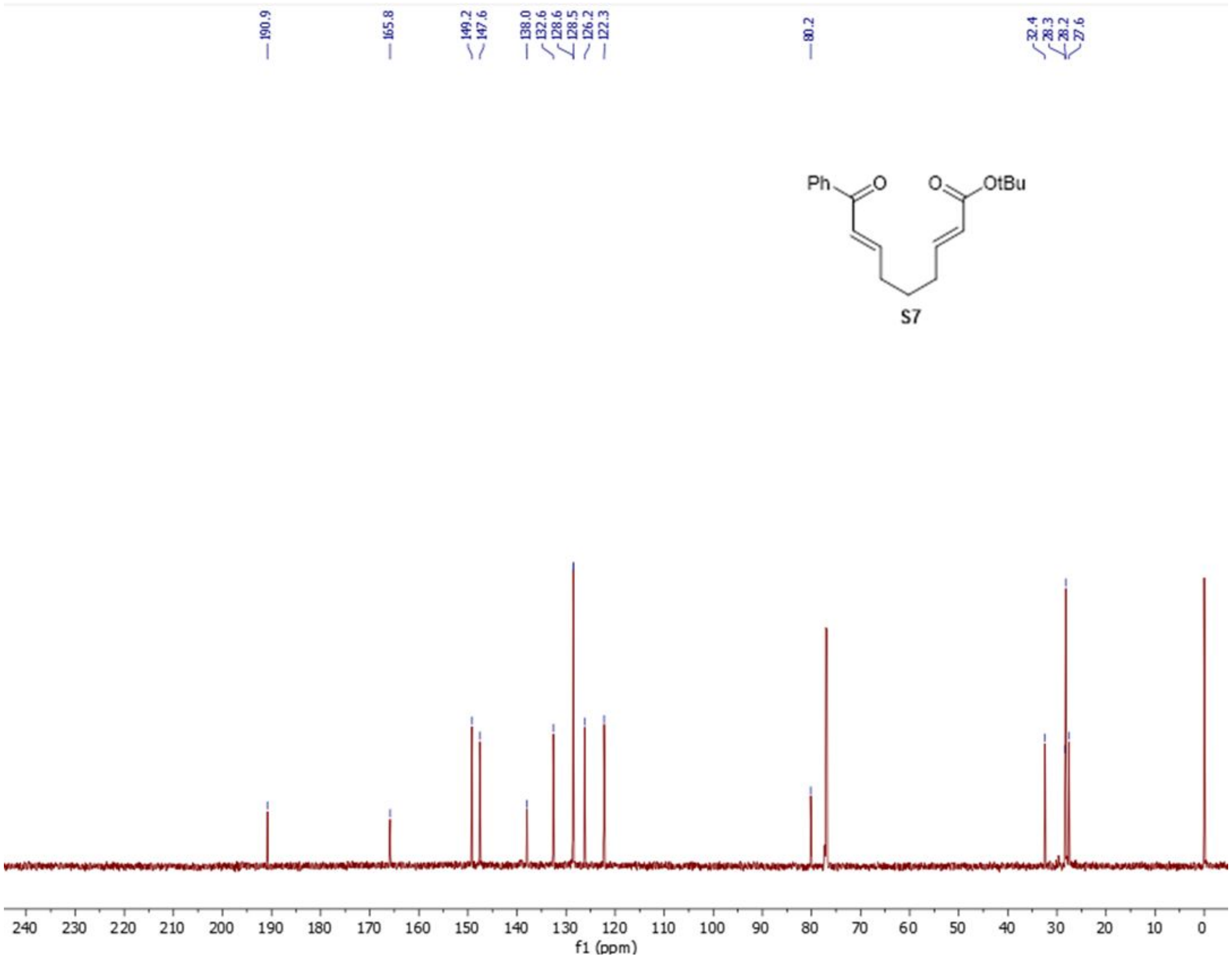

Figure 13. $^{13}C\{^{1}H\}$ NMR for compound **S7** (600 MHz, $CDCl_3$)

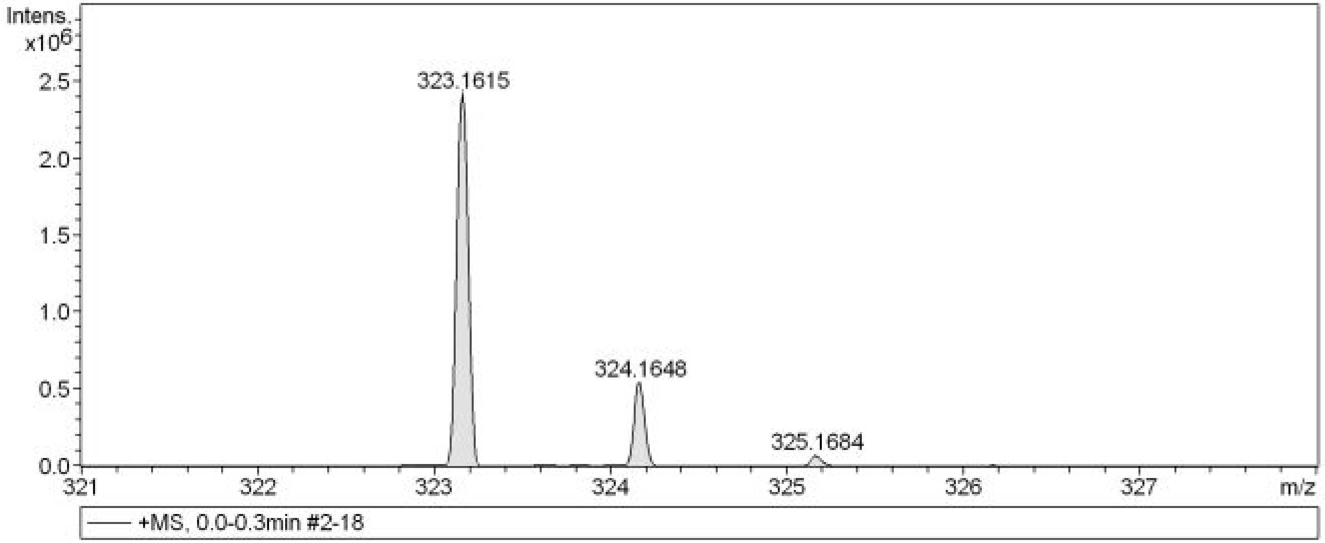

Figure 14. HRMS spectra for compound **S7**

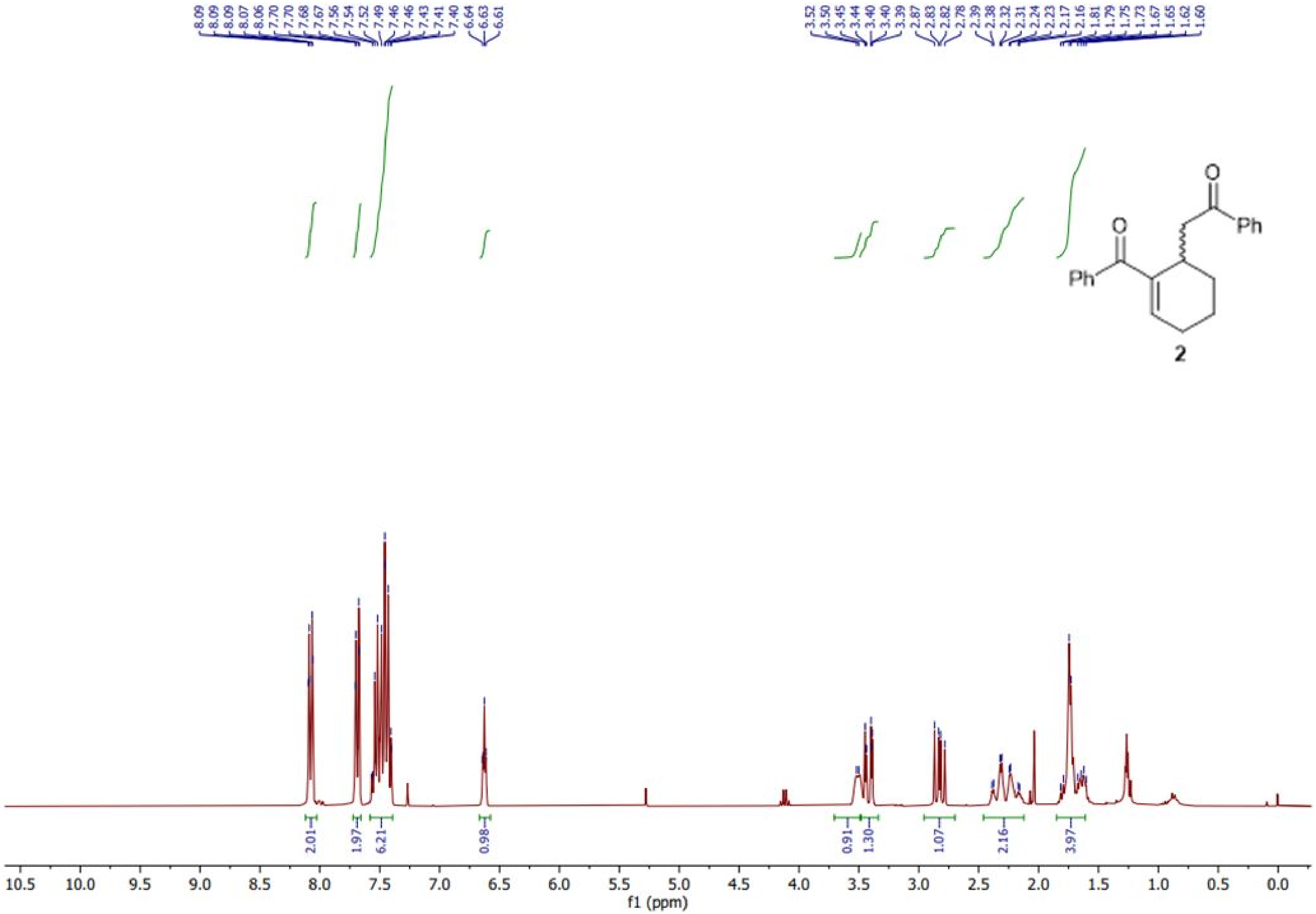

Figure 15. ^{1}H NMR for compound **2** (600 MHz, $CDCl_3$)

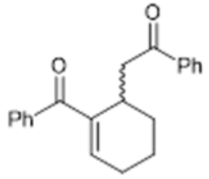

2

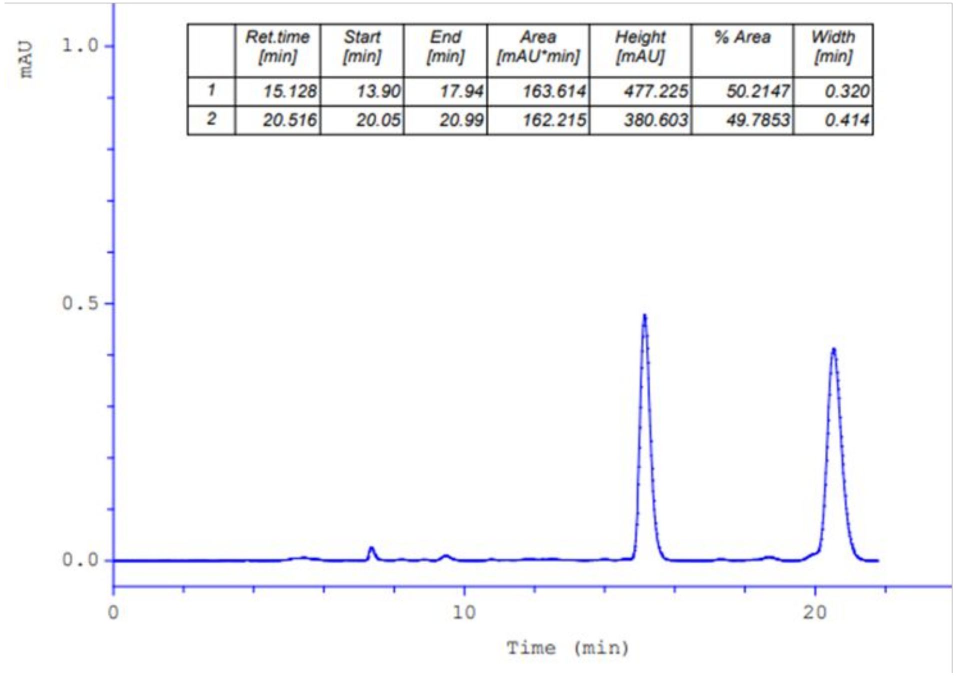

	Ret.time [min]	Start [min]	End [min]	Area [mAU*min]	Height [mAU]	% Area	Width [min]
1	15.128	13.90	17.94	163.614	477.225	50.2147	0.320
2	20.516	20.05	20.99	162.215	380.603	49.7853	0.414

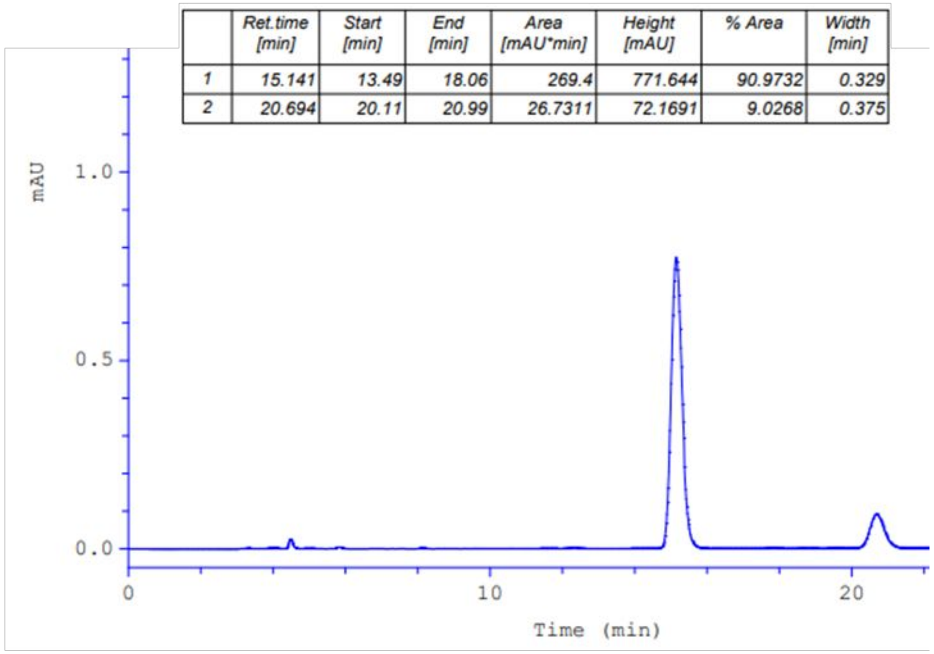

	Ret.time [min]	Start [min]	End [min]	Area [mAU*min]	Height [mAU]	% Area	Width [min]
1	15.141	13.49	18.06	269.4	771.644	90.9732	0.329
2	20.694	20.11	20.99	26.7311	72.1691	9.0268	0.375

Figure 16. HPLC Chromatogram for compound **2**

(Chiracel AD-H, 96:4 hexane: 2-propanol, 21 °C, flow 0.5 mL/min)

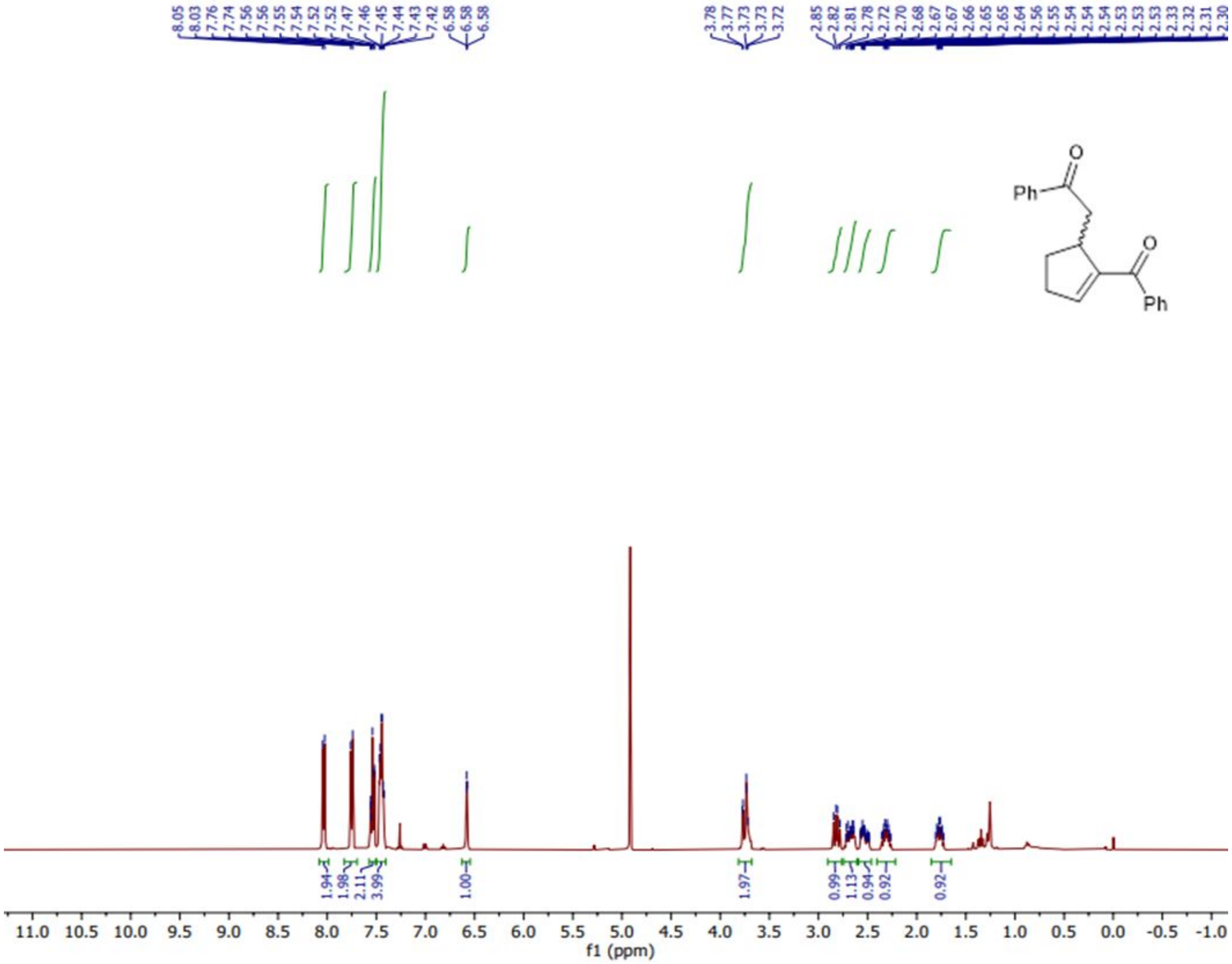

Figure 17. ^{1}H NMR for compound **10**(300 MHz, $CDCl_3$)

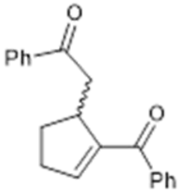

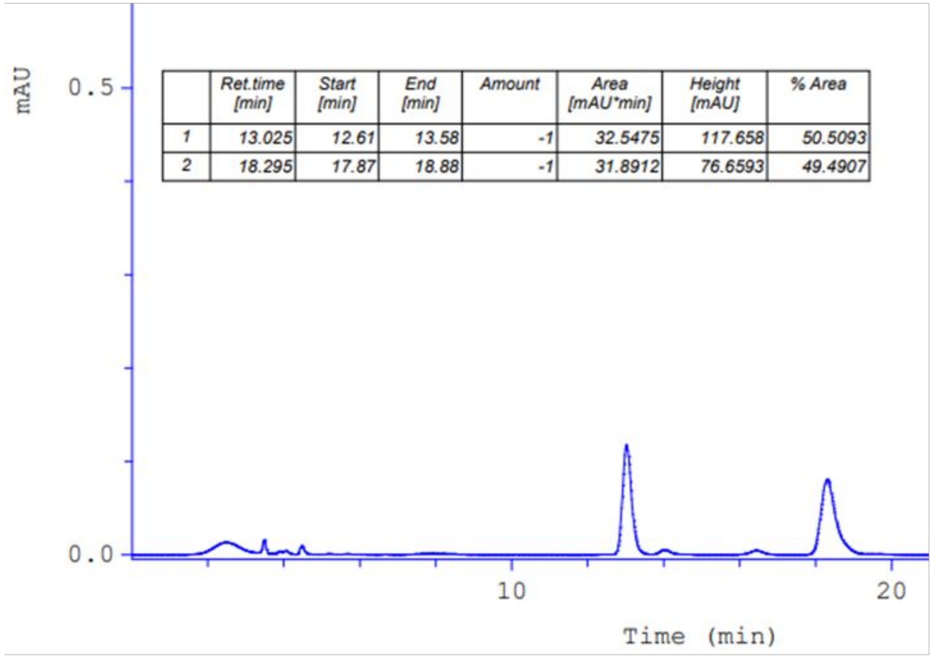

	Ret.time [min]	Start [min]	End [min]	Amount	Area [mAU*min]	Height [mAU]	% Area
1	13.025	12.61	13.58	-1	32.5475	117.658	50.5093
2	18.295	17.87	18.88	-1	31.8912	76.6593	49.4907

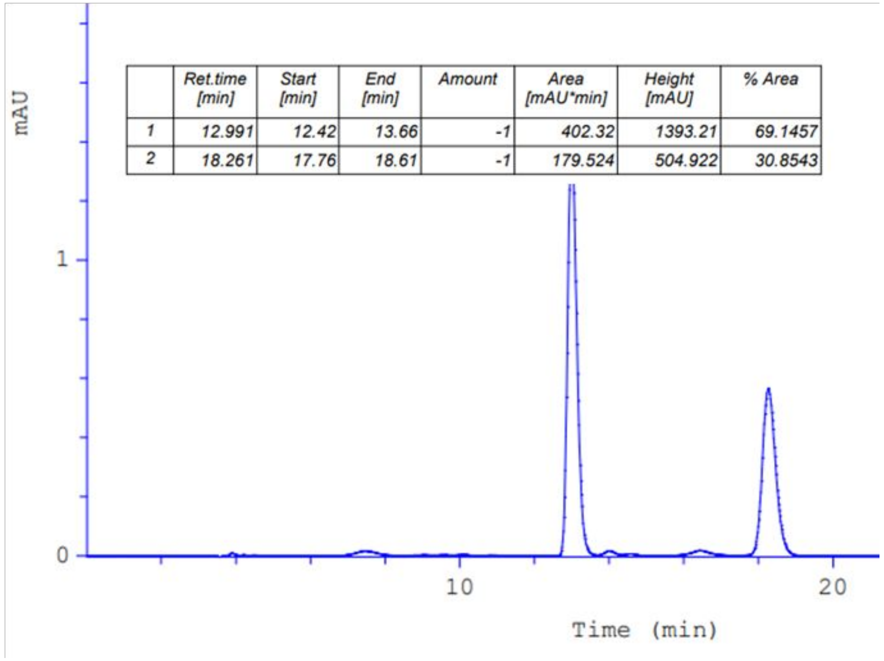

	Ret.time [min]	Start [min]	End [min]	Amount	Area [mAU*min]	Height [mAU]	% Area
1	12.991	12.42	13.66	-1	402.32	1393.21	69.1457
2	18.261	17.76	18.61	-1	179.524	504.922	30.8543

Figure 18. HPLC Chromatogram for compound **10**

(Chiracel AD-H, 96:4 hexane: 2-propanol, 21 °C, flow 0.5 mL/min)

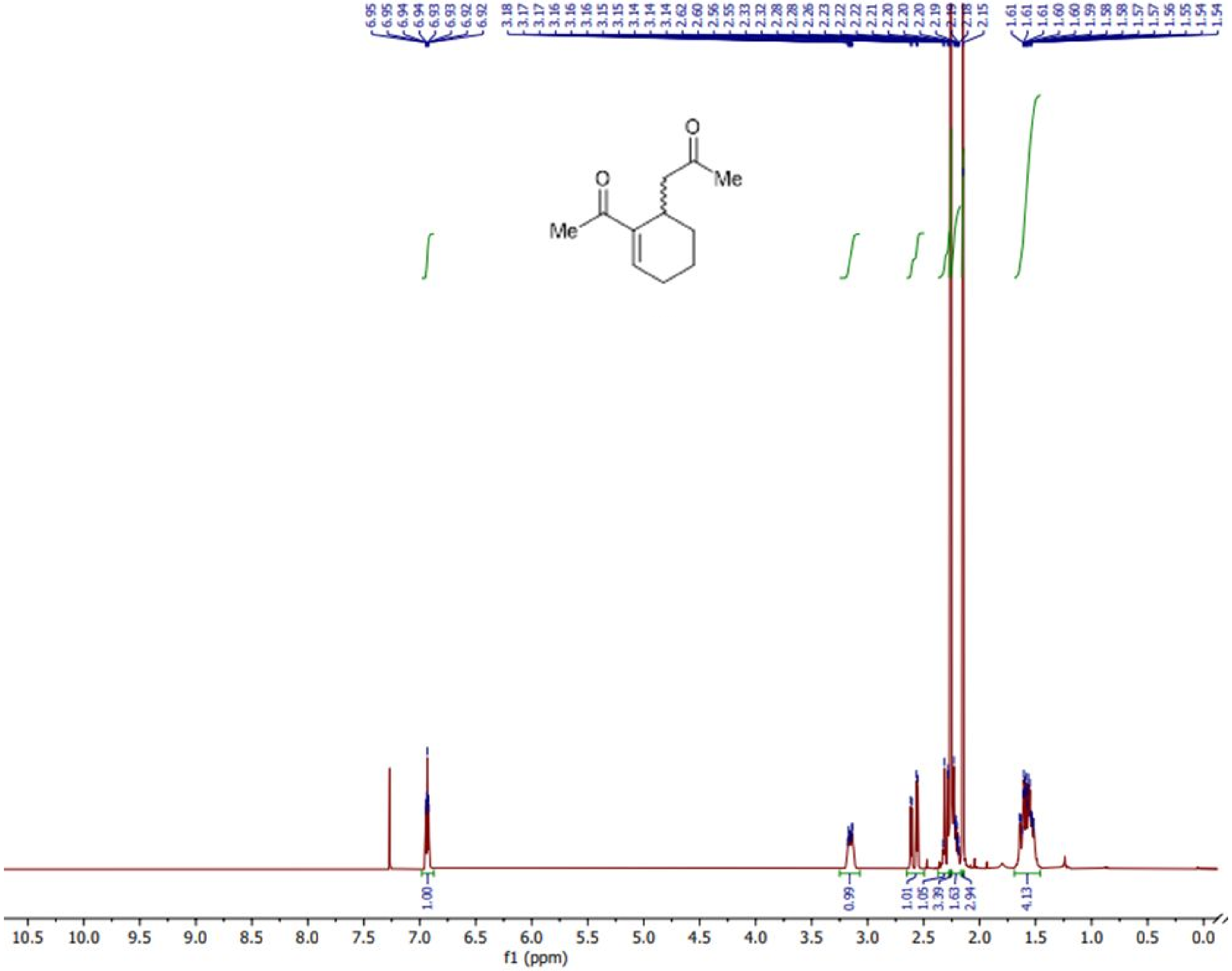

Figure 19. ^{1}H NMR for compound **11** (600 MHz, $CDCl_3$)

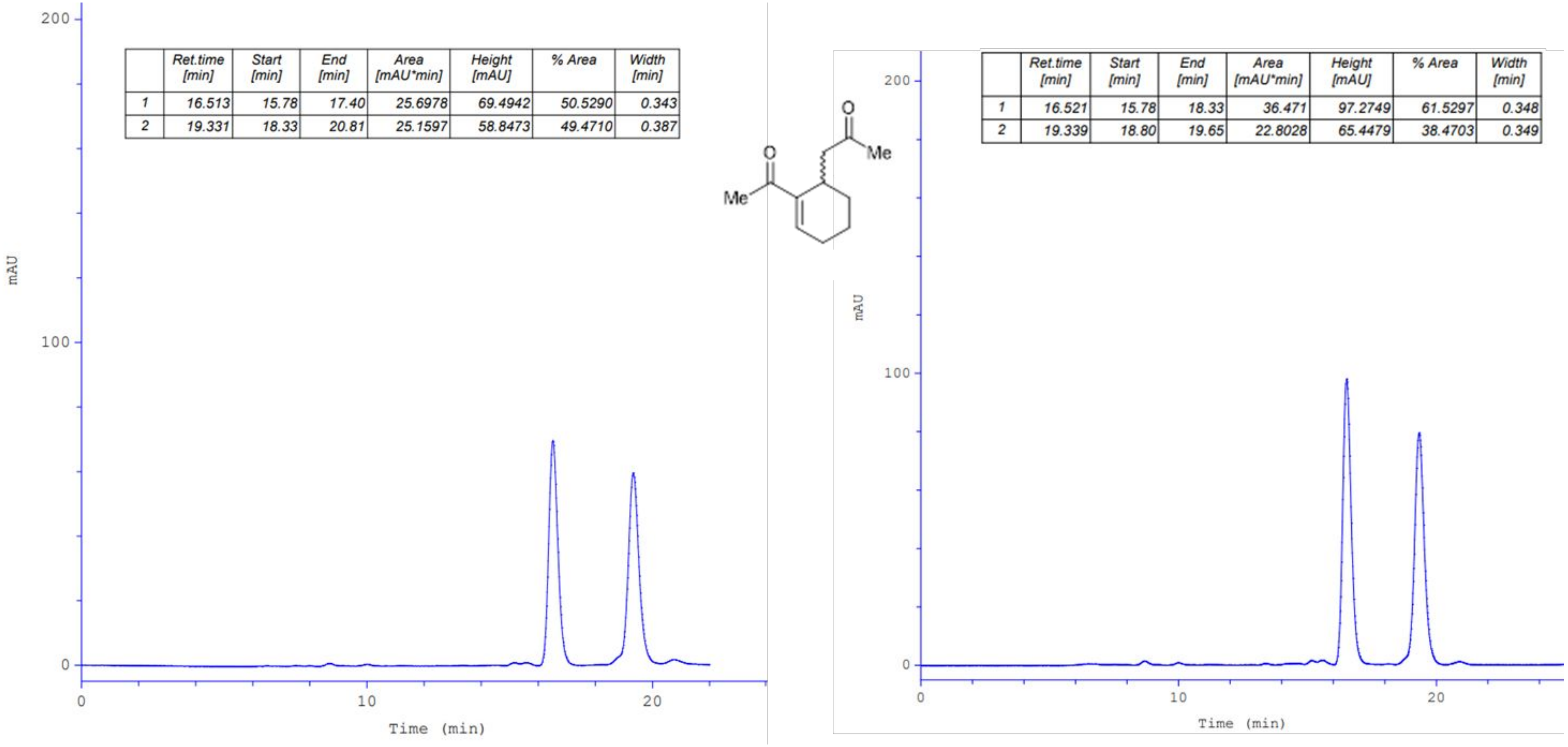

	Ret.time [min]	Start [min]	End [min]	Area [mAU*min]	Height [mAU]	% Area	Width [min]
1	16.513	15.78	17.40	25.6978	69.4942	50.5290	0.343
2	19.331	18.33	20.81	25.1597	58.8473	49.4710	0.387

	Ret.time [min]	Start [min]	End [min]	Area [mAU*min]	Height [mAU]	% Area	Width [min]
1	16.521	15.78	18.33	36.471	97.2749	61.5297	0.348
2	19.339	18.80	19.65	22.8028	65.4479	38.4703	0.349

Figure 20. HPLC Chromatogram for compound **11**

(Chiracel AD-H, 96:4 hexane: 2-propanol, 21 °C, flow 0.5 mL/min)

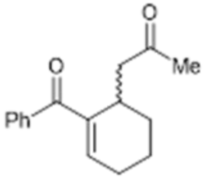

	Ret.time [min]	Start [min]	End [min]	Area [mAU*min]	Height [mAU]	% Area	Width [min]
1	21.027	20.10	22.09	787.378	1481.42	49.2733	0.490
2	28.464	27.55	29.50	810.602	1192.65	50.7267	0.640

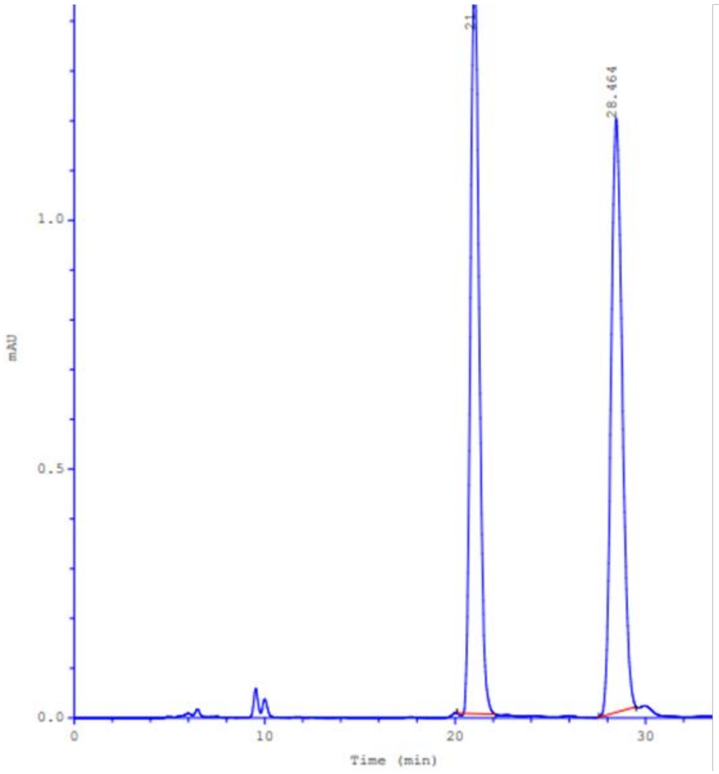

	Ret.time [min]	Start [min]	End [min]	Area [mAU*min]	Height [mAU]	% Area	Width [min]
1	21.024	20.38	22.38	1128.81	2152.62	49.5657	0.478
2	28.460	27.60	29.64	1148.59	1747.33	50.4343	0.619

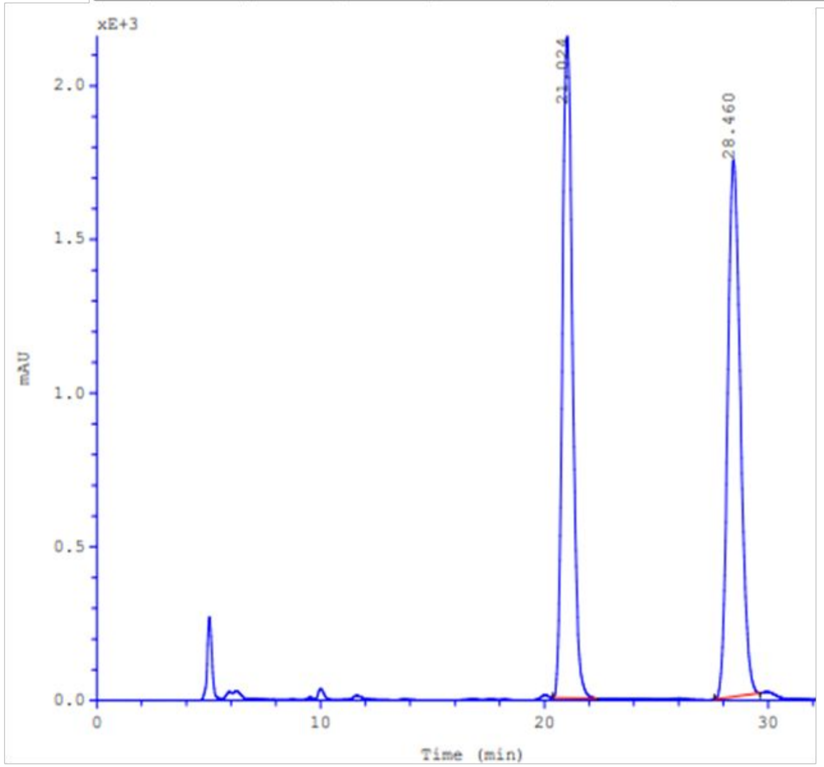

Figure 21. HPLC Chromatogram for compound **12**

(Chiracel AD-H, 96:4 hexane: 2-propanol, 21 °C, flow 0.5 mL/min)

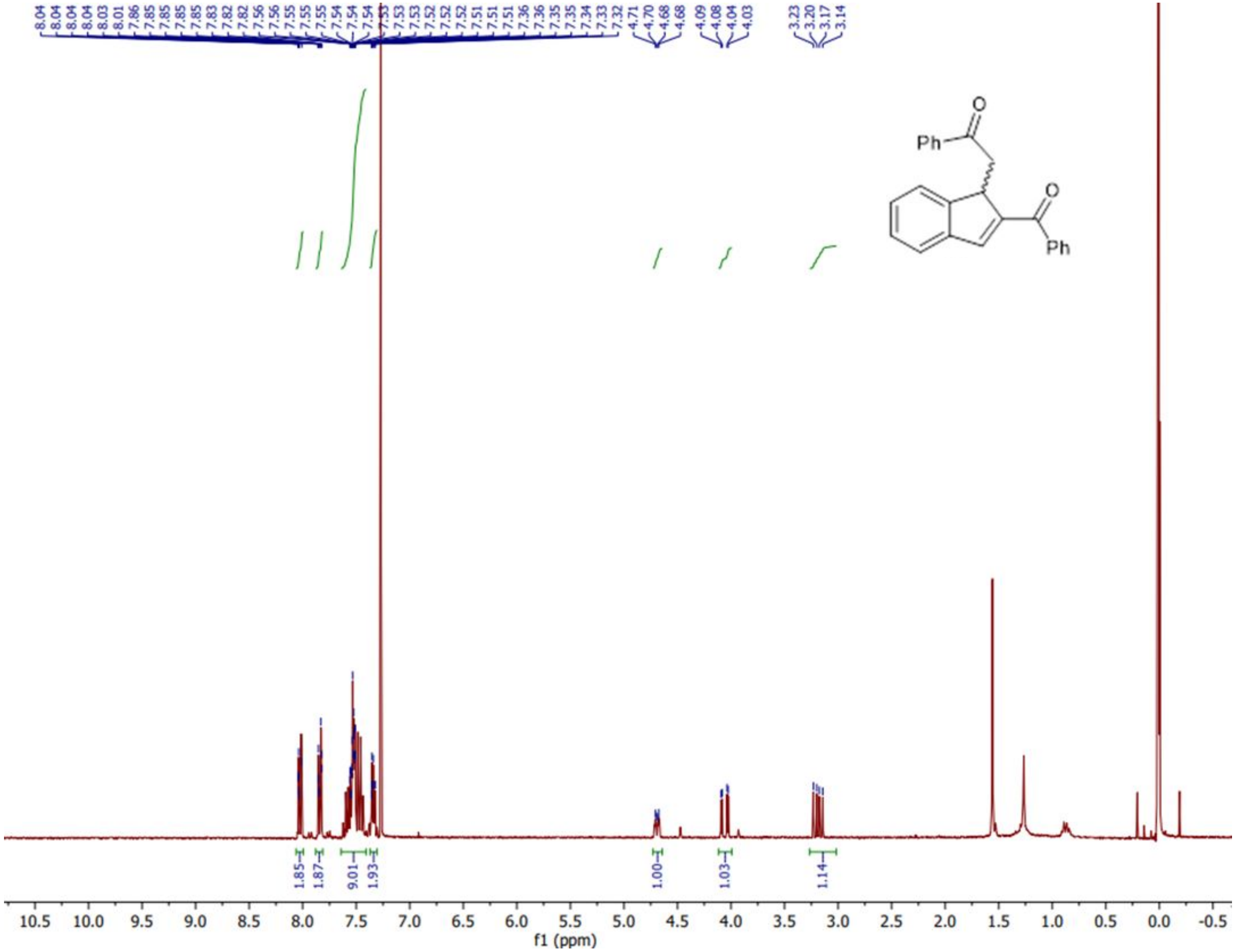

Figure 22. ^{1}H NMR for compound **13** (600 MHz, $CDCl_3$)

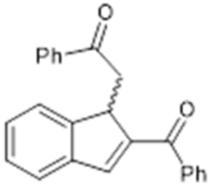

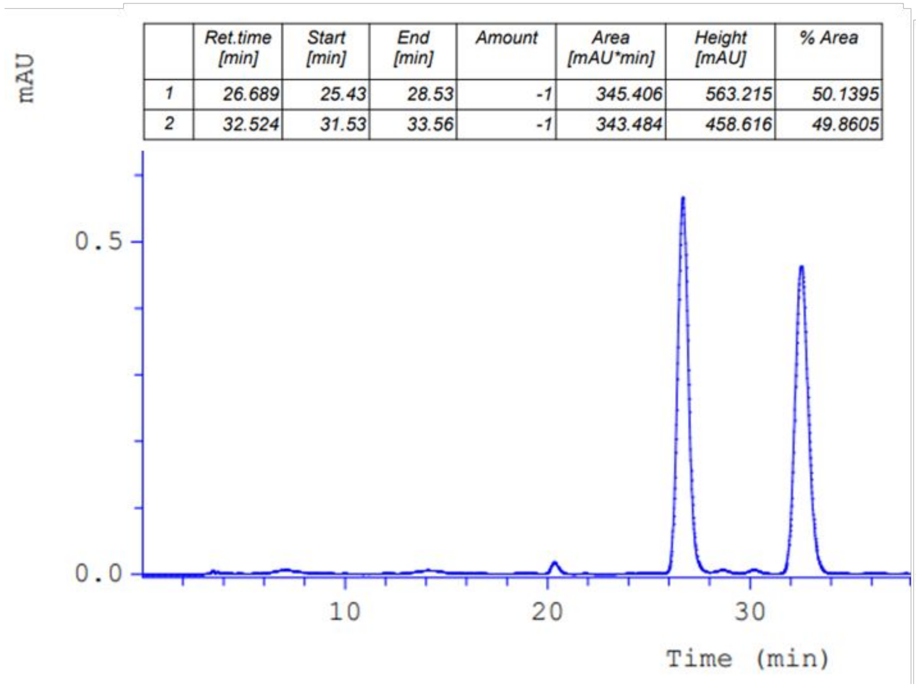

	Ret.time [min]	Start [min]	End [min]	Amount	Area [mAU*min]	Height [mAU]	% Area
1	26.689	25.43	28.53	-1	345.406	563.215	50.1395
2	32.524	31.53	33.56	-1	343.484	458.616	49.8605

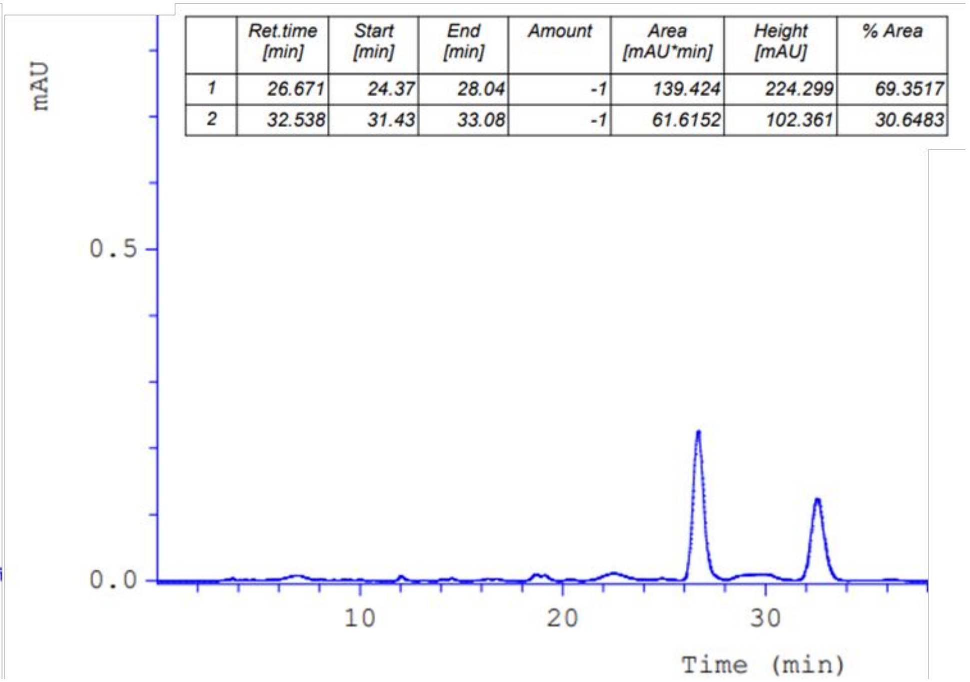

	Ret.time [min]	Start [min]	End [min]	Amount	Area [mAU*min]	Height [mAU]	% Area
1	26.671	24.37	28.04	-1	139.424	224.299	69.3517
2	32.538	31.43	33.08	-1	61.6152	102.361	30.6483

Figure 23. HPLC Chromatogram for compound **13**

(Chiracel AD-H, 96:4 hexane: 2-propanol, 21 °C, flow 0.5 mL/min)

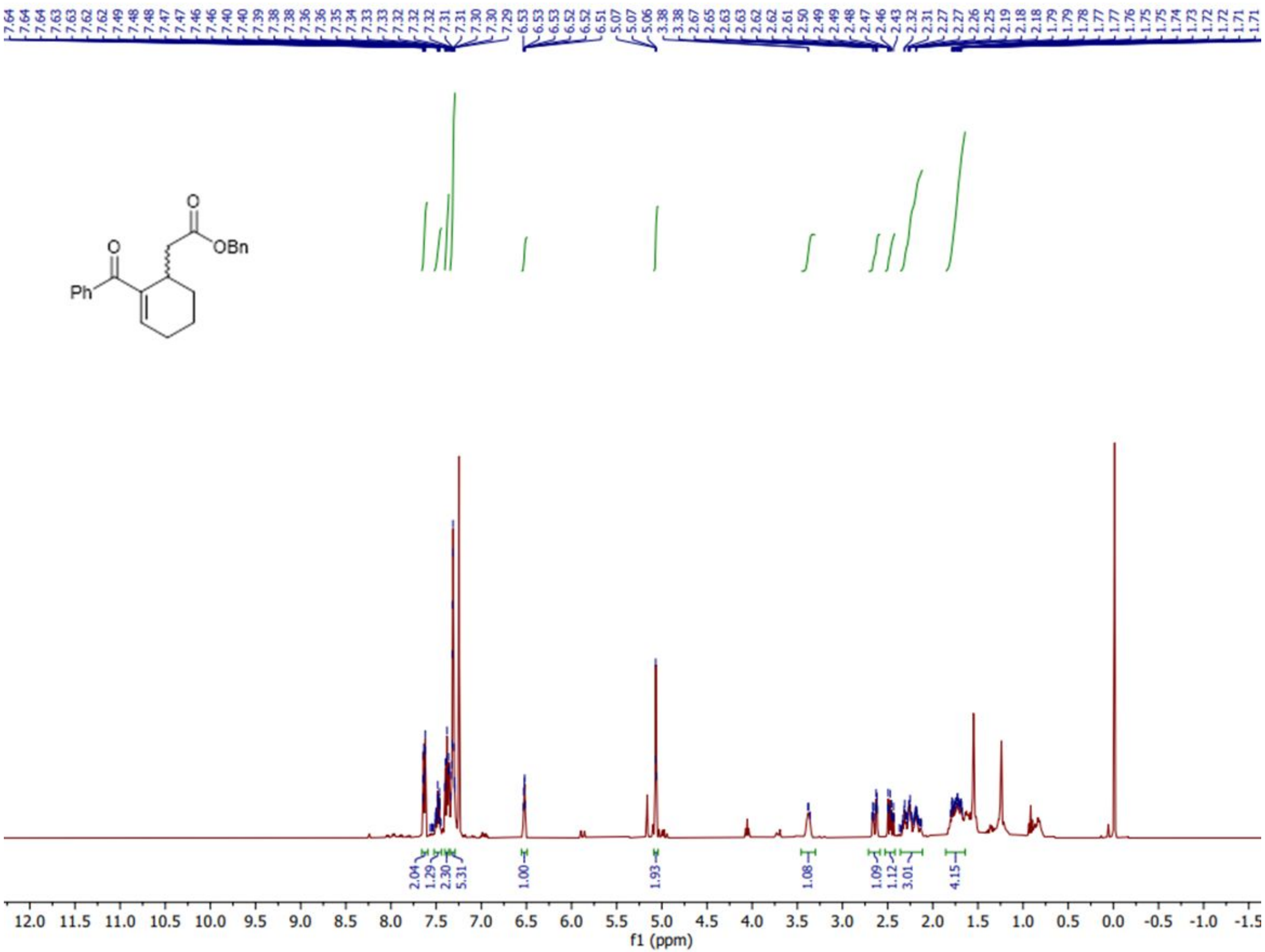

Figure 24. ^{1}H NMR for compound **14** (300 MHz, $CDCl_3$)

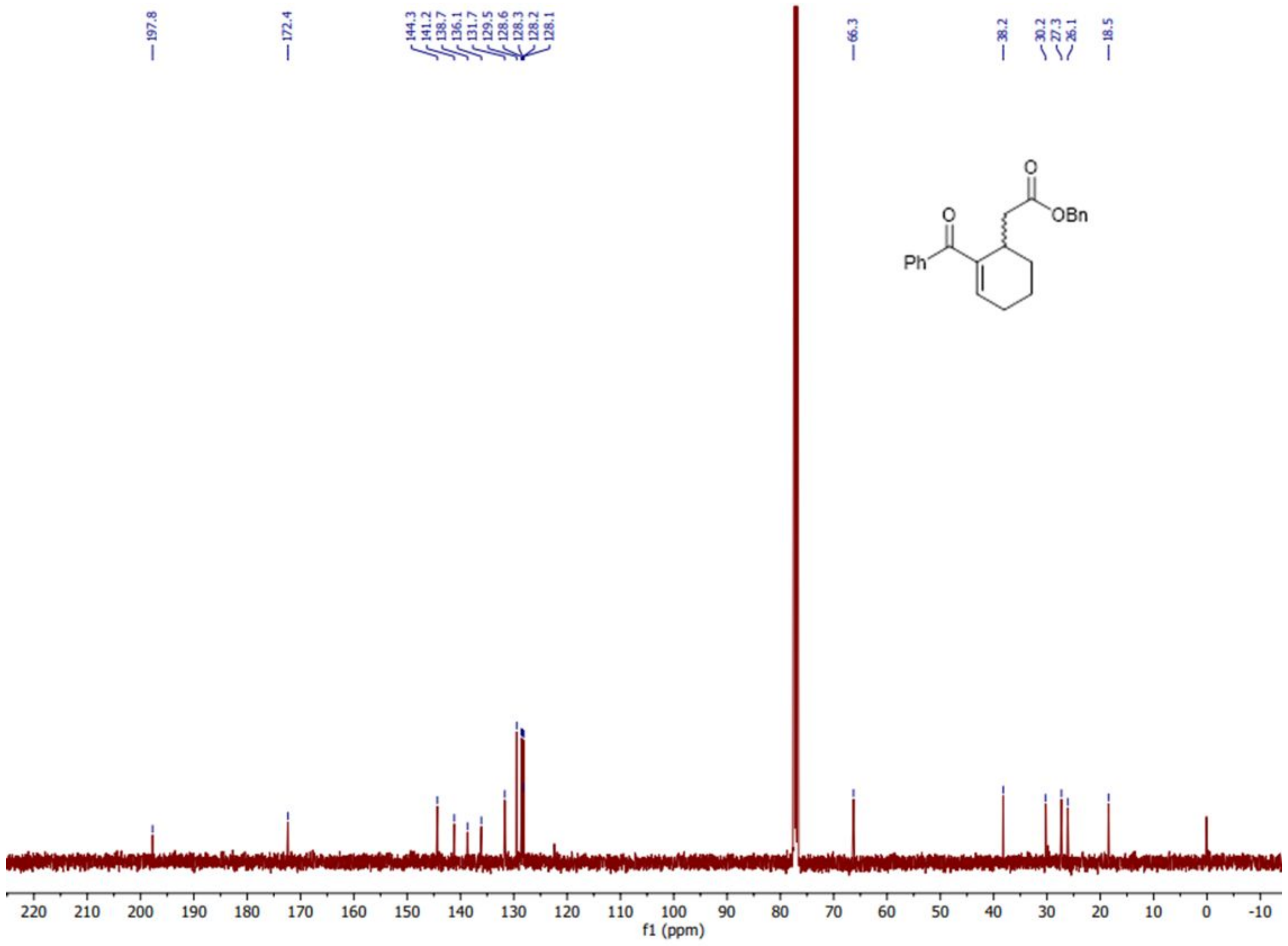

Figure 25. $^{13}C\{^{1}H\}$ NMR for compound **14** (300 MHz, $CDCl_3$)

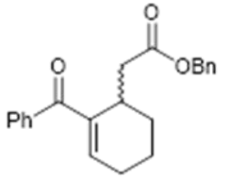

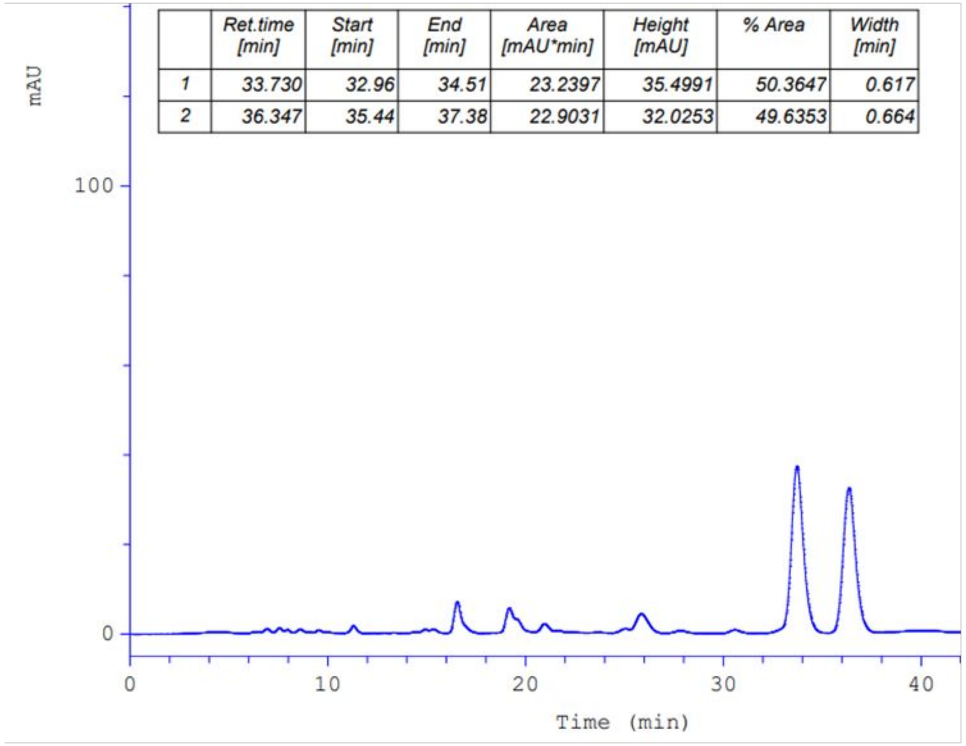

	Ret.time [min]	Start [min]	End [min]	Area [mAU*min]	Height [mAU]	% Area	Width [min]
1	33.730	32.96	34.51	23.2397	35.4991	50.3647	0.617
2	36.347	35.44	37.38	22.9031	32.0253	49.6353	0.664

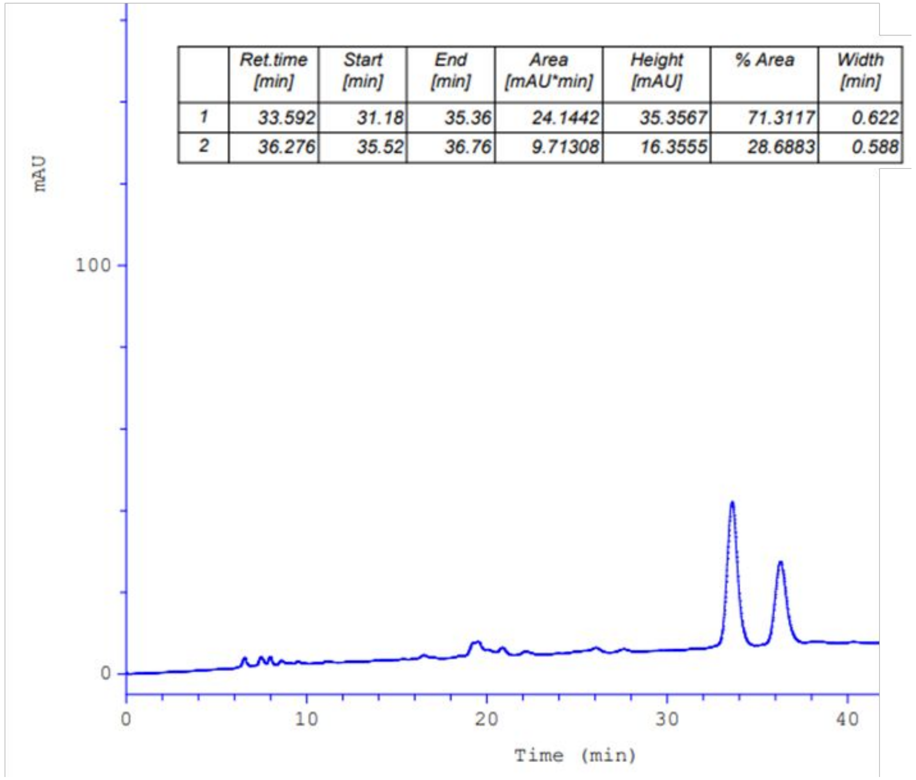

	Ret.time [min]	Start [min]	End [min]	Area [mAU*min]	Height [mAU]	% Area	Width [min]
1	33.592	31.18	35.36	24.1442	35.3567	71.3117	0.622
2	36.276	35.52	36.76	9.71308	16.3555	28.6883	0.588

Figure 26. HPLC Chromatogram for compound **14**

(Chiracel AD-H, 96:4 hexane: 2-propanol, 21 °C, flow 0.5 mL/min)

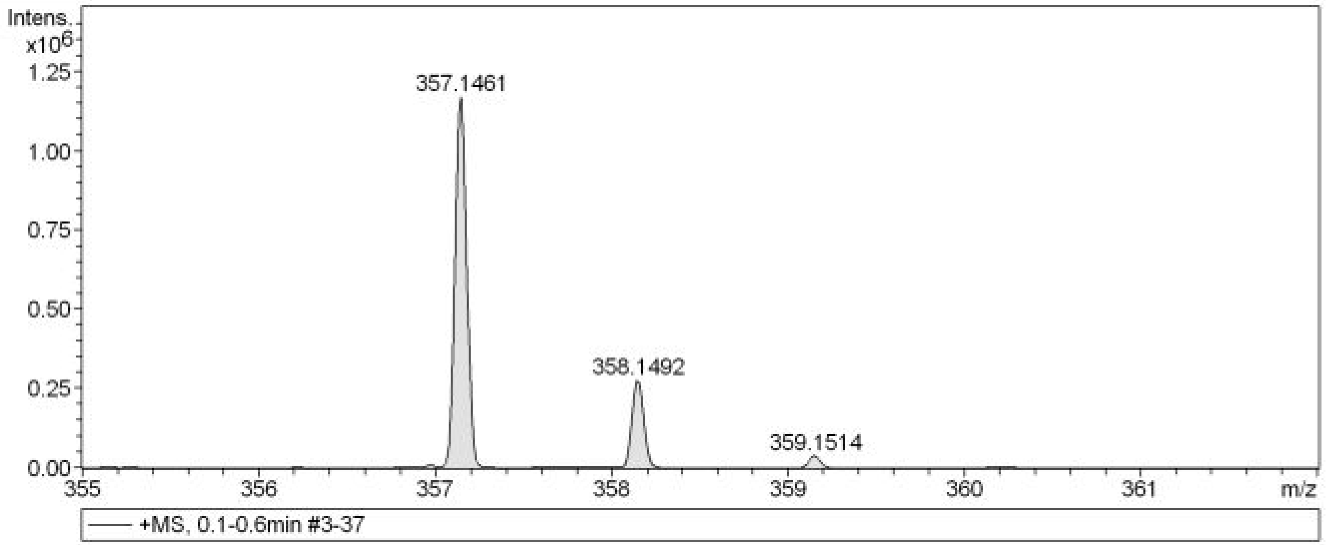

Figure 27. HRMS spectra for compound **14**

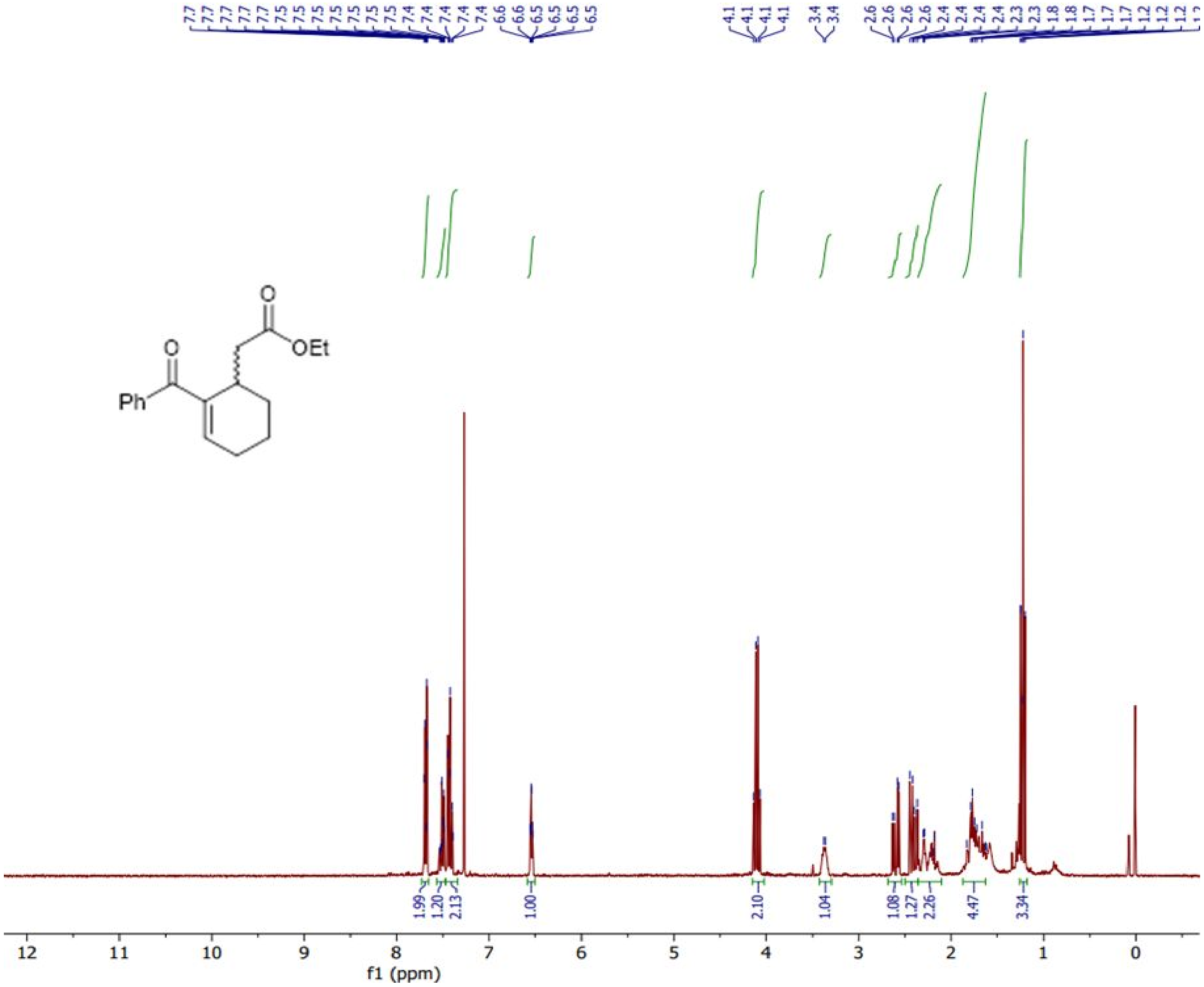

Figure 28. ^{1}H NMR for compound **15** (300 MHz, $CDCl_3$)

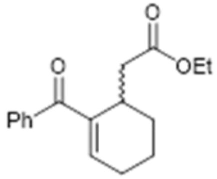

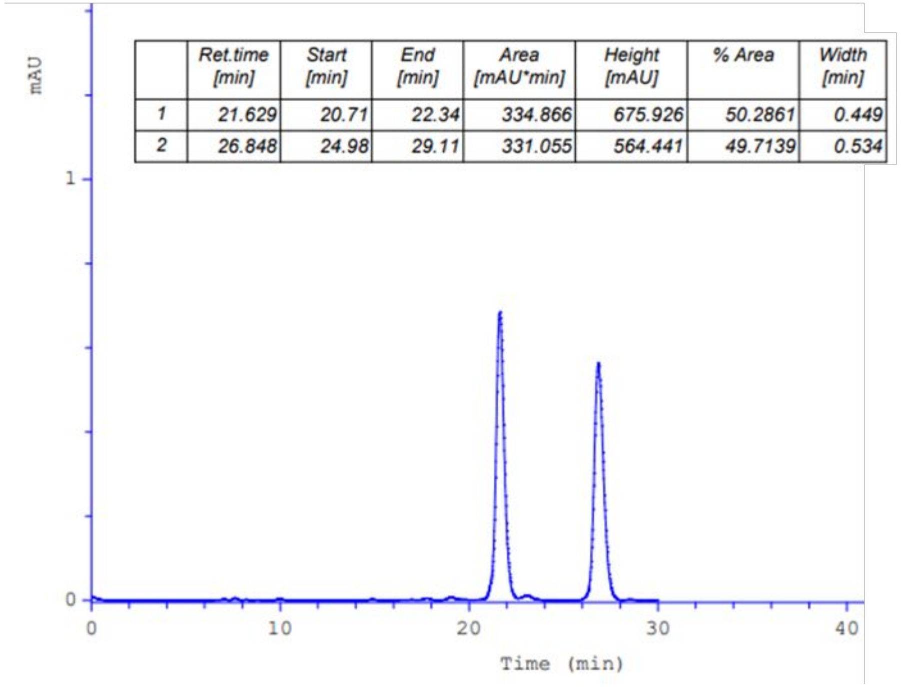

	Ret.time [min]	Start [min]	End [min]	Area [mAU*min]	Height [mAU]	% Area	Width [min]
1	21.629	20.71	22.34	334.866	675.926	50.2861	0.449
2	26.848	24.98	29.11	331.055	564.441	49.7139	0.534

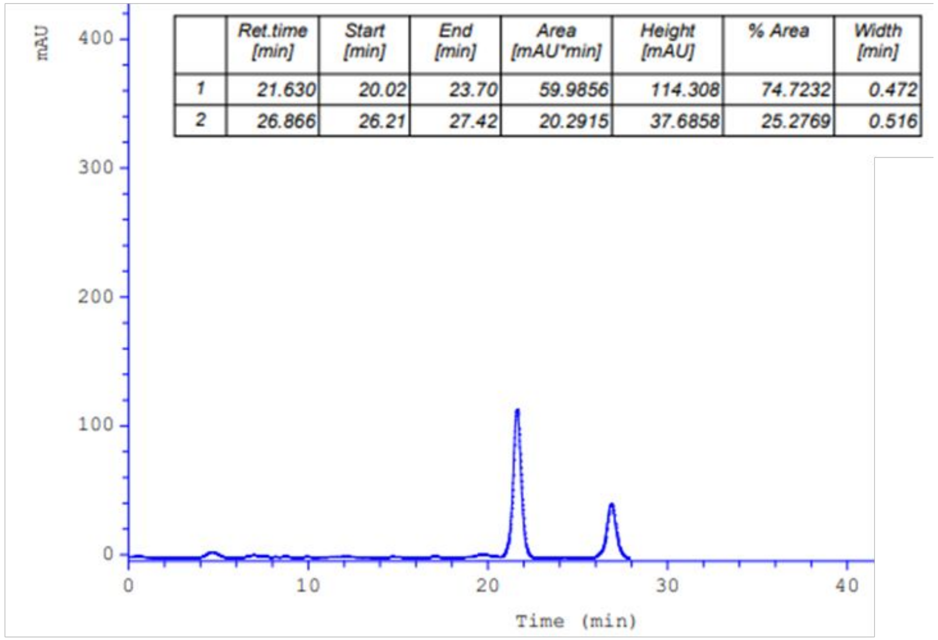

	Ret.time [min]	Start [min]	End [min]	Area [mAU*min]	Height [mAU]	% Area	Width [min]
1	21.630	20.02	23.70	59.9856	114.308	74.7232	0.472
2	26.866	26.21	27.42	20.2915	37.6858	25.2769	0.516

Figure 29. HPLC Chromatogram for compound **15**

(Chiracel AD-H, 96:4 hexane: 2-propanol, 21 °C, flow 0.5 mL/min)
